# The act of detecting a stimulus contaminates measures of conscious experience with decision biases

**DOI:** 10.1038/s41467-026-72567-6

**Published:** 2026-05-08

**Authors:** Nicolás Sánchez-Fuenzalida, Chris Jungerius, Stephen M. Fleming, Simon van Gaal, Johannes J. Fahrenfort

**Affiliations:** 1 Department of Psychology, University of Amsterdam, Amsterdam, The Netherlands; 2 Amsterdam Brain & Cognition, University of Amsterdam, Amsterdam, The Netherlands; 3 Department of Applied and Experimental Psychology, Free University Amsterdam, Amsterdam, The Netherlands; 4 Institute for Brain and Behavior Amsterdam, Free University Amsterdam, Amsterdam, The Netherlands; 5 Swammerdam Institute for Life Sciences, University of Amsterdam, Amsterdam, The Netherlands; 6 Institute of Cognitive Neuroscience and Department of Experimental Psychology, University College London, London, United Kingdom; 7 Max Planck-UCL Centre for Computational Psychiatry and Ageing Research, University College London, London, United Kingdom

**Keywords:** Human behaviour, Human behaviour

## Abstract

A central challenge in consciousness research is determining whether observers have a conscious experience of a stimulus. However, present/absent detection judgments are often biased by contextual factors, making it difficult to isolate conscious perception from non-perceptual influences. Traditional psychophysical methods struggle to disentangle these components. To address this, we conducted in-person experiments (N = 505) in which participants detected and reproduced dim and absent contrast-defined Gabor stimuli under three contextual manipulations: attentional cues, asymmetrical base rates, and payoff schemes. Using a reproduction task together with a Hurdle-Gaussian model, we quantitatively decomposed reproduction responses into a perceptual continuous contrast component and a non-perceptual “hurdle” component. We found that statistical priors (base rate) and reward structures (payoff) induced non-perceptual shifts in the reproduction hurdle, whereas attentional cues selectively shifted the continuous contrast component, consistent with changes in conscious experience. Critically, comparing conditions with and without intermixed detection trials revealed that the presence of a detection task contaminates reproduction reports with non-perceptual criterion effects. This highlights the need for caution in using and interpreting results that rely on detection judgments, even when combined with subjective measures like reproduction, especially given the central role that detection tasks play in consciousness research.

## Abstract (web summary)

A central challenge in consciousness research is determining whether people truly experience a stimulus. Here, the authors show that context biases traditional detection measures, whereas continuous reproduction better reflects conscious experience.

## Introduction

It is well-known that decisional processes can be influenced both by perceptual and non-perceptual factors. A central challenge in consciousness science is therefore to determine whether a particular behavioral profile stems from changes in the conscious experience of the observer, or from non-perceptual sources – such as decision biases^1,2^. Consider the following example: a person sitting in a dark room is instructed to detect a recurring faint light that appears on a screen by pressing a button. After some time, the experimenter imposes a significant monetary penalty for each missed light. As a result, the person now reports the light more frequently. However, his sensitivity is unaffected, as both the number of hits and the number of false alarms increases. Does the observer in this example consciously perceive a greater number of lights after the introduction of the penalty, or does he simply press the button more often – without any concomitant change in his conscious experience? In this example, it is not immediately clear how to assess whether the increased number of reported lights stems from a change in the observer’s conscious experience or from a non-perceptual change in the decision process intended to maximize rewards. When this question was posed to a group of consciousness researchers, 2 out of 3 respondents indicated that they did not think the observer would subjectively experience the light more often, while 1 out of 4 indicated that this would definitely cause a change in conscious experience^3^.

An empirical resolution to this debate remains elusive, partly due to limited progress in developing psychophysical methods to disentangle non-perceptual biases from perceptual effects. The need for a principled method to distinguish between changes in conscious perception from effects on cognitive or response biases is particularly urgent when we consider that a cornerstone method for studying (un)conscious perception is to contrast trials in which an observer is aware of the stimulus against trials where the observer reports no conscious experience (see refs. ^4,5^ for a review). Without being able to independently gauge the effects of experimental manipulations on subjective perception, such an approach conflates differences in conscious experience with response biases^6–11^. Unfortunately, Signal Detection Theory (SDT) models that separate perceptual sensitivity (d’) from bias^12^ cannot distinguish between these options^1,13^, as biases can be both perceptual and non-perceptual in nature. For example, in visual illusions, contextual factors may shift the observer’s percept (e.g., make the percept seem larger or smaller) compared to the “ground truth” that the experimenter may have adopted by performing direct measurements of the stimulus on screen or on paper^14–16^. Depending on what is considered a “correct” response in this setting, changes in decision criterion may reflect either perceptual or non-perceptual effects (see ref. ^17^ for a more in-depth characterization of this problem).

Here, we tackle this problem by harnessing “reproduction” measures, in which observers are asked to reproduce their experience of the stimulus directly^1,2^. Reproduction measures have a long history in psychophysics (for instance, underpinning the classical matching studies used to identify trichromacy in color vision), but have gained less traction in consciousness science. The rationale for using a reproduction task in the present study is that, unlike explicit detection reports, reproduction does not require participants to adopt a binary presence/absence decision criterion. We reasoned that this would allow us to sidestep decision biases related to decision criterion placement^6^ and to obtain a more direct estimate of the perceived stimulus strength (contrast in our case). In previous work we have successfully used reproduction measures to disentangle experimental manipulations that alter perception and those that are non-perceptual in nature^1,2^.

At the same time, it is important to note that reproduction measures are not completely bias-free either, despite their sensitivity to graded perceptual effects. Indeed, reproduction measures can be susceptible to multiple post-perceptual influences such as memory-related biases, category boundary effects, regression to the mean, and other decision-level factors (e.g^18,19^). A well-studied example is the effect of trial history, or serial dependency in perceptual decision making about stimulus orientation^20,21^. A growing body of literature suggests that serial dependence reflects a mixture of perceptual and post-perceptual effects caused by integration with previous decisions^22–27^. We add to this list by showing that randomly intermixing a detection task with a contrast reproduction task in the same experimental block induces strong non-perceptual effects on reproduction responses (Experiment 1 and 2), while removing the detection task also removes these non-perceptual effects on reproduction (Experiment 3). In the discussion, we return to the post-perceptual influence of detection judgments on contrast reproduction and the implication of this finding for consciousness research.

As a general approach, we asked observers to either detect dim stimuli (detection task) or to directly reproduce the perceived contrast of contrast-defined near‑threshold Gabor stimuli (reproduction task). We used three bias manipulations to manipulate observers’ relative proportion of ‘seen’ and ‘not-seen’ responses: an exogenous attentional cue which has previously been shown to affect perceived contrast^28^, as well as an asymmetrical base rate and a pay-off scheme. Based on our previous work, we expected the detection task to exhibit substantial decision biases, while the reproduction task, which is less directly tied to an explicit presence/absence criterion, would better allow for the isolation of effects on subjective experience. With this approach, our goal was twofold: (1) to determine whether these commonly used contextual manipulations influence subjective experience or instead reflect changes in decision processes, and (2) to demonstrate how the reproduction task can be used to assay conscious perception in an experiment that contains both stimulus-present and stimulus-absent trials.

Foreshadowing the results, we confirm that all manipulations affected responses in both the detection task and in the reproduction task, but that these responses were influenced by different perceptual and non-perceptual sources depending on the manipulation. By conducting extensive computational modeling to tease apart these two effects, we conclude that the attentional manipulation consistently affected subjective experience, whereas the base rate and payoff manipulations did not. Crucially, we carry out a control experiment to uncover how the presence of a detection task (as opposed to a difference in base-rate alone) drives the emergence of non-perceptual biases in reproduction responses.

Finally, to be clear, we focus on a specific and relatively simple case: the detection/perception of near‑threshold Gabor patches with a fixed spatial frequency. Our conclusions therefore apply most directly to contrast‑defined stimuli in low‑dimensional feature space, where reproduction of a single feature (e.g., contrast) is feasible and well-defined. Extending our approach to more complex, high‑dimensional stimuli (such as objects) or to other sensory modalities is an important question for future work but lies beyond the scope of the present experiments. However, as we will show, our results demonstrate how general detection requirements can affect criterion-free subjective measures of conscious experience, such as reproduction tasks or other appearance-based measures^29^.

## Results

### Experiment 1: reproduction and detection

**Fig. 1:**
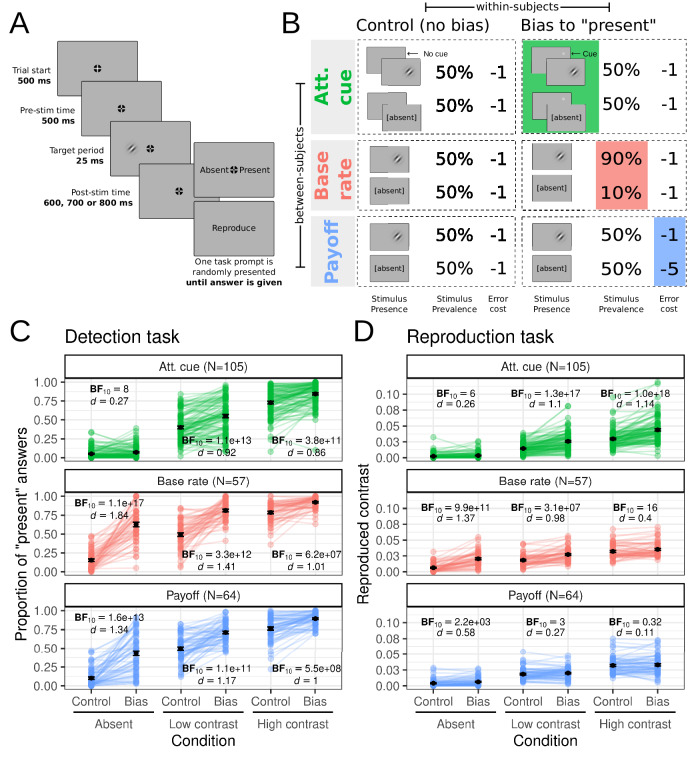
Experiment 1. Trial layout, bias manipulations summary and main results. **A** A trial consisted of an initial fixation period (1000 ms; the fixation was made following recommendations by Thaler and colleagues^83^ to reduce eye movements), followed by a target period (25 ms), a second fixation (600, 700 or 800 ms), and finally followed by the prompt of one of the two tasks (shown until a response was given). In the detection task, participants simply pressed a button for absence or presence. In the reproduction task, participants used the mouse wheel to increase or decrease contrast of a reproduction item that would appear for the same duration as the target item (25 ms). The reproduction item (re-)appeared with a frequency of 1 Hz while the contrast was adjusted by the observer. Crucially, participants did not know which task they were performing until after stimulus offset. **B** Observers completed the detection and reproduction task without any manipulation in place (control condition) or with one of three bias manipulations: attentional cue, payoff or base rate. In the attentional cue condition, a small circle was presented for 66.67 ms shortly before the start of the target period (ISI 50 ms; compare with ref. ^28^). The cue was presented both in absent and present trials and therefore could not be used to predict presence or absence of the target, but it would always appear on the same side as the target in present trials. In the base rate condition 90% of the trials contained a target. In the payoff condition, five points were deducted for misses, whereas false alarms only cost one point. **C** Detection task results. The proportion of ‘present’ responses of each participant along with the group average for each bias source and condition. **D** Reproduction task results. The average contrast reproduction (Michelson contrast) of each participant along with the group average for each bias source and condition. All error bars indicate the SEM. All BF values correspond to a paired one-sided t-test with a Cauchy prior (centered around zero with a width of 0.707), d values indicate Cohen’s d effect size.

We asked participants to detect (detection task) or to reproduce (reproduction task) a dim Gabor patch, also referred to as the target patch. On each trial, either a Gabor patch was presented at low or high contrast (present trials), or no patch was presented (absent trials). At the end of every trial, a prompt indicated whether they had to make a present/absent decision or give a reproduction response. Importantly, participants gave only one response type on each trial, either detection or reproduction. In each block, on 50% of the trials a detection response had to be given, and on the other 50% a reproduction response (randomly intermixed). On detection trials, the text *Absent - Present* appeared centered on the screen until participants answered by pressing the left (to answer “absent”) or right (to answer “present”) button of the mouse. On reproduction trials, the word *Reproduce* appeared on the screen until the participant used the mouse wheel to adjust the contrast of the reproduction patch. In order to reproduce their subjective experience, participants had to adjust the contrast of the reproduction patch to match their experience of the target patch (reproduction task; see Fig. 1A for a graphical depiction of the trial layout). The detection and reproduction task were randomly interleaved. Further, each participant was assigned to one of three between-subjects contextual manipulations: (i) a condition in which a small non-predictive cue was presented just before the target period (attentional cue condition), (ii) a condition containing nine times more present than absent trials (base rate condition) and (iii) a condition in which misses cost five times more than false alarms (payoff condition). Each of these three biased conditions was accompanied by a within-subjects neutral control condition (see Fig. 1B for a graphical summary of the manipulations). Note that although the attentional cue would always appear on the same side of the target, it was non-predictive cue because it would equally often appear on target-present and on target-absent trials. See “Methods” for a more detailed description of the manipulations and sample sizes.

To assess the effect of the manipulations on the detection task, we first calculated the proportion of ‘present’ responses for each contrast level, condition and participant (see Fig. 1C). Overall, all manipulations resulted in positive effects, such that observers answered ‘present’ more often when any of the bias manipulation were in place, relative to the control condition, both in absent and present trials. All manipulations had a large effect on the proportion of present responses across all contrast levels (all Cohen’s *d* > 0.8), except for the attentional cue on absent trials where the effect was smaller (*d* = 0.27). A Bayesian t-test showed decisive evidence for an effect compared to the null in all conditions (BF_10_ > 100), except for absent trials in the attentional cue condition, where the evidence for an effect was moderate (BF_10_ = 8; all Bayesian t-tests are paired and one-sided with a Cauchy prior centered around 0, with a width of .707 unless stated otherwise; the exact Cohen’s *d* and Bayes Factor values for each comparison can be found in Fig. 1C).

Next, to determine whether these changes in decision criterion also resulted in changes in subjective experience, we calculated the mean reproduced contrast for each contrast level, condition and participant (see Fig. 1D). The first thing to note is that participants were able to successfully reproduce the ordered relationship between three contrast levels, with the lowest average reproduced for absent trials, slightly higher for low contrast trials, and even higher for high contrast trials. Further, when the attentional cue was presented, observers reproduced patches with higher contrast as compared with their responses in the accompanying control condition. This was true both for stimulus-absent (*d* = 0.26) and present trials (*d* ≥ 1.1). A Bayesian t-test revealed moderate evidence for an effect in absent trials (BF_10_ = 6) and decisive evidence for an effect in present trials compared to the null (all BF_10_ > 100). In the base rate condition, reproductions were higher across all contrast levels when stimulus-present trials were more likely (*d* ≥ 0.4, BF_10_ ≥ 16). Finally, in the payoff condition, reproductions were higher when misses were punished more than false alarms in absent (*d* = 0.58, BF_10_ = 2.2e + 03) and in low contrast trials (*d* = .27, BF_10_ = 3), but there was no effect in high contrast trials (*d* = .11, BF_10_ = 0.32). To summarize, an increase in reproduced contrast was more likely than a null effect in all conditions across all contrast levels, except for high contrast trials in the payoff condition.

Together, these results suggest that all manipulations result in large decision biases. Further, the attentional cue and base rate conditions also show clear corresponding changes in reproduced contrast across all levels (absent, low and high contrast). In contrast, the payoff manipulation selectively increased the perceived contrast of absent and low contrast trials. Taking the reproduction measure at face value, one might be tempted to conclude that all manipulations not only have a strong influence on decision bias but also on conscious experience (i.e., reproduction) at nearly all contrast levels. However, an increase in the average reproduced contrast as reported in Fig. 1D may in fact result from two distinct influences on reproduction behavior: on the one hand, the overall average reproduced contrast reflects the reproduced contrast on trials in which participants made the reproduction patch visible (a reproduction with a higher-than-zero contrast; i.e., a “present” reproduction), but on the other hand, it also reflects the ratio of present to absent reproductions (i.e., reproductions with zero contrast). From here onwards we refer to present reproductions (reproductions with a higher-than-zero contrast) as *non-zero* contrast reproductions and to absent reproductions as *zero* reproductions. In both cases the *non-zero* and *zero* values in question refer to the contrast level of the reproduction measure.

**Fig. 2:**
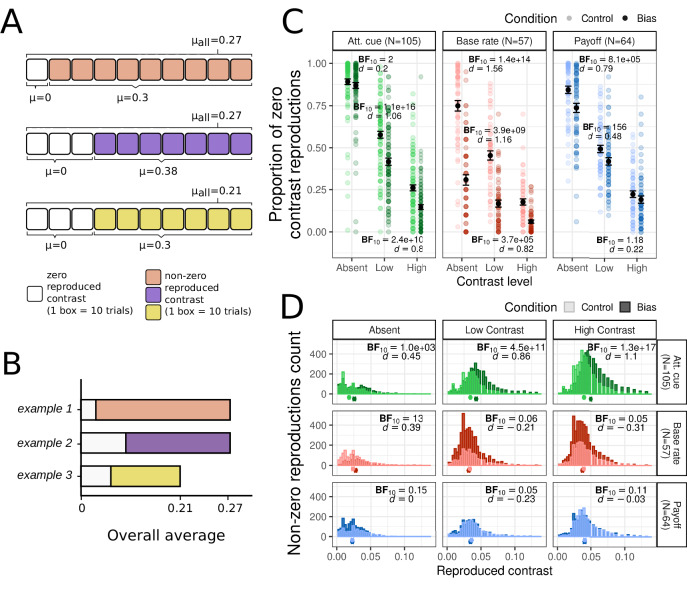
Experiment 1 Reproduction Results. **A**, **B** Schematic representation of how the proportion of zero reproductions (zero contrast reproductions, or “absent reproduction”) as well as the average contrast of non-zero reproductions (higher-than-zero contrast reproduction, or “present reproduction”) can independently influence the overall average reproduced contrast. **C** Average proportion of zero reproductions per participant and condition. **D** Distribution of non-zero reproductions. All the reproduction responses of all participants are plotted as a histogram. The average reproduced contrast for each histogram is plotted as a dot below the distribution. Note that some participants could not be plotted because they did not have any non-zero reproduction on absent trials; 11 participants in the cue condition, 2 in the base rate condition and 8 in the payoff condition. All error bars indicate the SEM. All BF values correspond to a paired one-sided t-test with a Cauchy prior (centered around zero with a width of 0.707), d values indicate Cohen’s d effect size.

To clarify the role of the distinct influences on reproduction behavior, consider for example the situation in Fig. 2A, B. In this example an observer reproduces 10 patches as having *zero* contrast, and 90 patches as having *non-zero* contrast, with an average *non-zero* contrast of 0.3. The overall average reproduced contrast is then 0.27. Let us now consider a situation where the same observer reproduces 30 *zero* contrast patches, and 70 *non-zero* contrast patches with an average reproduced contrast of 0.38. Although both the average reproduced contrast of *non-zero* patches is different from the first example (0.3 in the first, 0.38 in the second) and the ratio of *zero* to *non-zero* reproductions is also different (1:9 in the first, 3:7 in the second), the overall average reproduced contrast would still be 0.27, because the overall average reproduced contrast is affected both by the average reproduced contrast of *non-zero* reproductions and by the ratio of *zero* to *non-zero* reproductions. Similarly, consider a third situation in which the average *non-zero* reproduction contrast is 0.3, just as in the first example, but now the ratio of *zero* to *non-zero* reproductions is 3:7 as in the second example. The overall reproduced contrast is now different from that in both examples (0.21 rather than 0.27), despite having the same average reproduced contrast as in the first example and the same ratio of *zero* to *non-zero* reproductions as in the second example.

These examples illustrate how both the proportion of *zero* reproductions and the average contrast of *non-zero* reproductions (Fig. 2A) together independently contribute to the overall average reproduced contrast (Fig. 2B). This reflects a limitation of the reproduction measure. It is possible that subjects make an initial binary decision–present or absent–and use the zero-contrast rating to indicate “absent” on trials where they are asked to reproduce, despite having a non-zero contrast experience. Because this proportion of present/absent decisions will be affected by the same non-perceptual biases that affect binary responding, it is not surprising that we might also see it reflected in the proportion of zero-contrast responses. To tease apart these two putative influences on the overall average reproduced contrast in Fig. 1D, we conducted an exploratory analysis in which we split the reproduction data into zero and non-zero contrast reproductions and complement the results of this approach with extensive computational modeling. We subsequently validated the results and approach in an independent follow-up experiment which we outline further below (Experiment 2).

First, we assessed how often observers reproduced a *zero* contrast patch by calculating the proportion of *zero* reproductions per condition, participant and contrast level (see Fig. 2C). Overall, the proportion of *zero* reproductions decreased as the contrast of the stimuli increased, but also when any of the bias manipulations were in place. A Bayesian t-test revealed decisive evidence (*d* > 0.4 BF_10_ > 100) of an effect in all conditions across all contrast levels, except for absent trials of the attentional cue condition and high contrast trials of the payoff condition, where there was anecdotal evidence for an effect (BF_10_ < 3). This shows that all manipulations increased the probability of reporting a *non-zero* contrast patch, echoing the result of the manipulations on overall reproduced contrast in Fig. 1D.

Next, to assess the effect of the manipulations on *non-zero* reproductions, we first removed all the *zero* reproductions (Fig. 2D, see Supplementary Fig. S1 for the distributions prior to removing the zero-reproductions). Interestingly, the reproduction data without *zero* reproductions in Fig. 2D yields a rather different pattern of results compared to those in Figs. 1D and 2C. The attentional cue continued to result in strong evidence and medium to large effects across all contrast levels (*d* > 0.4, BF_10_ > 100). In the base rate condition however, although there was still strong evidence (BF_10_ = 13, *d* = 0.39) for a small effect in absent trials, there was now strong evidence against an effect in low (BF_10_ = 0.06, *d* = −0.21) and high (BF_10_ = 0.05, *d* = −0.31) contrast trials. Similarly, the payoff condition now showed moderate to strong evidence against an effect in absent (BF_10_ = 0.15, *d* = 0), low (BF_10_ = 0.05, *d* = −0.23) and high (BF_10_ = 0.11, *d* = −0.03) contrast trials.

This pattern of results can readily be explained by Fig. 2A: despite a different overall reproduced mean when *zero* reproductions are included (as in Fig. 1D), the reproduced mean on *non-zero* reproductions may remain largely the same when this bias is accounted for (payoff and base rate in Fig. 2D). This supports an inference that two different factors contribute to an overall increase in reproduced contrast (Fig. 1D). On the one hand, all manipulations result in an increase in the proportion of *non-zero* reproductions (see Fig. 2C) – equivalent to a bias towards binary “present” responses. However, for those patches reproduced with above-zero contrast, effects of contextual manipulations were largely restricted to the attentional cue (see Fig. 2D).

**Fig. 3:**
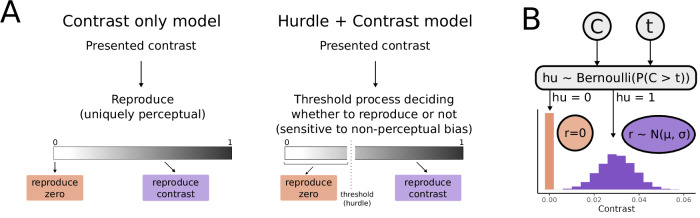
Reproduction task generative models. **A** Diagram of two potential generative models during the reproduction task. In the “Contrast only” model, participants always reproduce what they perceive. In the “Hurdle + Contrast model”, sensory information must first cross a threshold to be reproduced as a non-zero value (much like in a detection task). If the contrast of a patch is higher than some reproduction threshold (the reproduction hurdle) the observer reproduces what they perceived. However, if the contrast is lower than this threshold, the observer assumes the perceived input was noise and the reproduction patch is set to zero contrast. **B** Simplified schematic depiction of the Hurdle-Gaussian model, in which C represents the contrast of a target patch and t represents the reproduction hurdle of an observer. hu is then the binary outcome of a Bernoulli trial that depends on the probability of the contrast of the target patch C passing the reproduction hurdle t. When the contrast does not pass the threshold (hu=0) the reproduced contrast r is zero, otherwise r is drawn from a normal distribution centered on μ with a standard deviation of σ, where μ is a linear transformation of the stimulus contrast (for a detailed description of the model, see Supplementary Note S1).

To better understand these results, we hypothesized that the proportion of *zero* contrast reproductions may have a different origin than the average reproduced contrast of *non-zero* reproductions. One possibility is that the generative model that observers employ when they reproduce is not just a reproduction of the perceived contrast itself (Fig. 3A, left), but also a threshold process that determines whether a *non-zero* value should be reproduced or not (Fig. 3A, right). If this is correct, the reproduction task when intermixed with a detection task does not purely reflect what is subjectively perceived, but also partially reflects a threshold-based process, which as we describe above may be subject to non-perceptual influences. Together, these two processes indirectly influence the average reproduced contrast by changing the ratio of zero to *non-zero* reproductions.

In Fig. 2D we attempted to isolate the effect of *non-zero* reproductions by simply removing the reproductions with *zero* contrast. However, this procedure does not properly account for the separate effects of the threshold and contrast components on reproduced contrast. A more appropriate way of testing the presence of these two contributions is to fit a model that separately estimates a threshold on *zero* reproductions, as well as a parameter estimating the contrast for *non-zero* reproductions. A model that is well suited to achieve this is the so-called Hurdle-Gaussian model^30^, derived from a family of hurdle models that was originally developed in the field of econometrics^31^. This model is composed of a binary part (Bernoulli distribution) that predicts the probability of a particular contrast value passing a threshold that determines whether to reproduce or not (the hurdle parameter; from here on we refer to this threshold as the reproduction hurdle to prevent ambiguity with the decision threshold in the detection task), and a linear part that describes the distribution of the values that passed the threshold. See Fig. 3B for a graphical depiction of the model and compare to Supplementary Fig. S1 to see the visual resemblance between the model and the data it is intended to capture. Although not often used in psychology, hurdle models are much better suited to describe data with large numbers of zeroes compared to methods that rely on removing the zeroes before the analysis. Furthermore, they allow one to separately estimate whether a manipulation preferentially affects the probability of zero responses or whether it affects the distribution of the *non-zero* responses, which is our primary aim here^30^.

Therefore, to test whether the manipulations influenced the reproduction hurdle (i.e., the hurdle parameter), the contrast parameter, or both, we fit four hierarchical models to each bias source condition independently, allowing a random slope for each participant and condition (control and bias). We either allowed both the contrast and hurdle parameters to vary across conditions (full model), only contrast (contrast model), only hurdle (hurdle model) or we fixed both parameters across conditions (baseline model; see Supplementary Note S1 for a detailed description of the modeling procedure).

**Fig. 4:**
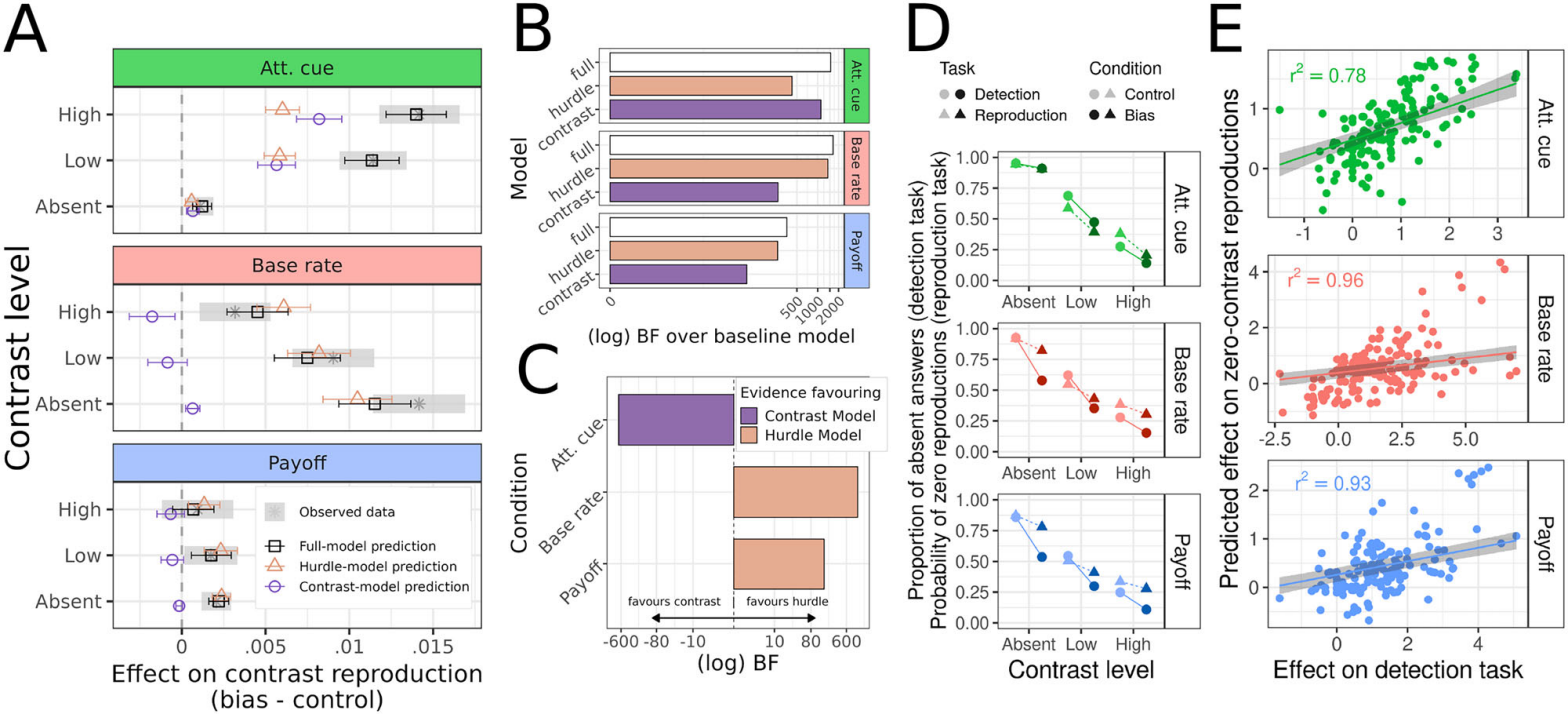
Experiment 1 Hurdle-Gaussian modeling. **A** Overall reproduction responses (including zero and non-zero contrast responses) predicted by the full model (hurdle and contrast effect; black squares), a contrast only model (purple circles), and a hurdle only model (salmon triangles, also see main text and Supplementary Note S1). Gray bars with asterisks indicate average observed overall reproduction difference between control and bias condition (as plotted in Fig. 1D). Error bars and gray bars indicate the 95% confidence interval. **B** Model comparison (log) Bayes Factor values. All models are compared against the baseline model (see main text). Positive values indicate preference for the model labeled in the y-axis, negative values indicate preference for the baseline model. **C** (log) Bayes Factor values of Model comparison between the hurdle and contrast models. Strength of the evidence favoring a model is plotted across the x-axis such that negative values indicate preference for the contrast model and positive values indicate preference for the hurdle model. **D** Proportion of “absent” responses in the detection task (circles with solid lines; cf. Figure 1C) compared to the probability of zero reproduction (i.e., not passing the hurdle) in the reproduction task (triangles with dashed lines) as predicted by the Hurdle-Gaussian model. **E** Linear regression between the observed effect (biased condition minus control condition) on the proportion of “absent” responses (logit-transformed) in the detection task and the model-predicted effect (biased condition minus control condition) on the probability of not passing the hurdle (logit-transformed) in the reproduction task. This analysis directly tests the relationship between the proportion of absent responses in the detection task and the model-predicted (Hurdle-Gaussian model) proportion of zero reproductions. The gray shaded area around the regression line corresponds to the 95% confidence interval.

Using the fitted parameters within each model, we sampled new reproduction responses to assess whether only contrast, only hurdle or both parameters were necessary to recover the original effect on overall reproduced contrast (see Fig. 4A). In the attentional cue condition only the full model correctly predicts the empirical overall reproduced contrast effects across all contrast levels. In contrast, in the base rate and payoff conditions, both the full and hurdle models are able to recreate the effect of the manipulations. This suggests that while the attentional cue seems to influence both the reproduction hurdle and reproduced contrast, the base rate and payoff effects are largely explained by a reproduction hurdle shift affecting the proportion of *zero* contrast reproductions. To formally test this claim, we compared all models against the baseline model and computed Bayes Factors for each comparison (see Fig. 4B). Bayes Factors were derived by estimating the marginal likelihoods of each model given the observed reproduction data^32^, which effectively estimates how well each model predicts the observed data. In all conditions, the full model was the best-performing model, suggesting all manipulations influenced both the reproduction hurdle and the reproduced contrast to some extent ((log) BF_full-over-baseline_ ≥ 300; (log) BF_full-over-second-best_ ≥ 90; note that BF values are reported in log scale due to their magnitude).

Next, to assess the relative contribution of the contrast and hurdle parameters, we directly compared the hurdle against the contrast model (see Fig. 4C). There was decisive evidence ((log) BF_contrast-over-hurdle_ = 687) in favor of the contrast model in the attentional cue condition, whereas the opposite was true in the payoff ((log) BF_hurdle-over-contrast_ = 168) and base rate ((log) BF_hurdle-over-contrast_ = 1133) conditions, suggesting that the attentional cue manipulation predominantly affects reproduced contrast, while the payoff and base rate conditions predominantly affect the reproduction hurdle.

Lastly, we reasoned that if the proportion of *zero* reproductions depends on the observer’s decision threshold in the detection task, then it should be highly correlated with the proportion of ‘absent’ responses in the detection task, as the latter directly indicates how often a patch is reported (see Fig. 4D for the proportion of absent responses in the detection task and the predicted probability of reproducing zero - i.e., not passing the hurdle - in the reproduction task as estimated by the Hurdle-Gaussian modeling on the reproduction task). To test this claim, we fitted a linear mixed model using the effect of each manipulation on the proportion of absent responses in the detection task to predict the probability of a zero reproduction that we obtained from the Hurdle-Gaussian model (see Fig. 4E; see Supplementary Note S2 for a detailed account of the analysis). For each bias manipulation there was decisive evidence (BF_10_ > 1000, *r*^2^ ≥ 0.7) for a positive association between the proportion of absent responses in the detection task and the predicted hurdle effect in the reproduction task, further confirming that the effect each manipulation had on altering the proportion of *zero* reproductions is most parsimoniously explained by a decision criterion shift in the detection task.

### Experiment 2: replication and modeling to disentangle sources of bias

Because the modeling results in Experiment 1 were exploratory, we wanted to independently replicate these results in a second experiment with planned modeling. Additionally, this allowed us to address some shortcomings of the original design. Notably, we had not accounted for some potential non-perceptual effects on reproduction in Experiment 1. For example, feedback on reproduction performance is known to produce post-perceptual effects on reproduction behavior, e.g. ^33^. Although feedback on the reproduction task was already uninformative in Experiment 1, we completely removed all feedback on the reproduction task in Experiments 2 and 3. Another potential post-perceptual influence on reproduction is response bias due to motor repetition. Reproducing the desired contrast level in Experiment 1 required the participants to consistently scroll the mouse wheel in the same direction on every trial. To minimize the influence of introducing an effort cost (and associated response biases) in the reproduction task in Experiments 2 and 3, we randomly varied the scrolling direction required to indicate higher contrast from trial to trial of the reproduction task. Finally, Experiment 1 contained a variable interval of 600, 700 or 800 ms between the presentation of the target and the cue to give a response. To minimize potential post-perceptual serial dependence effects on reproduction caused by temporal differences between trials, we replaced this with a fixed 600 ms interval in Experiment 2 and 3. See “Methods”*–General Procedure* and *Differences between Experiment 1, 2 and 3* for a detailed account of the precise changes. More broadly, we kept task demands and timing constant across all conditions in Experiments 2 and 3 to minimize the influence of potential confounding effects on experimentally relevant comparisons.

**Fig. 5:**
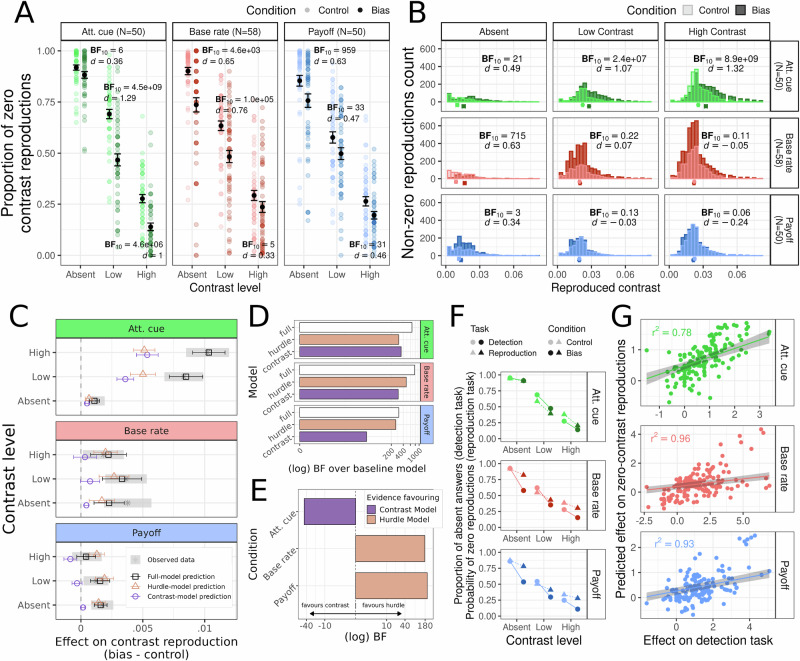
Experiment 2 results. **A** Average proportion of zero contrast reproductions per participant and condition. **B** Distribution of non-zero contrast reproductions. All the reproduction responses of all participants are plotted as a histogram (a dot below each histogram depicts the average). Note that some participants could not be plotted because they did not have any non-zero reproduction on absent trials; 8 participants in the cue condition, 10 in the base rate condition and 5 in the payoff condition. Error bars in (**A**) and (**B**) indicate SEM. **C** Overall reproduction responses (zero and non-zero contrast responses) predicted by the full model (hurdle and contrast effect, black squares), contrast only model (purple circles), and hurdle only model (salmon triangles). The gray asterisks indicate the average overall observed reproduction difference between control and bias condition (as plotted in Fig. 1D). Colored error bars and gray bar indicate the 95% confidence interval. **D** Model comparison (log) Bayes Factor values. All models are compared against the baseline model. Positive values indicate preference for the model labeled on the y-axis, negative values indicate preference for the baseline model. **E** (log) Bayes Factor values of Model comparison between the hurdle and contrast models. The evidence strength favoring a model is plotted (x-axis) such that negative values indicate preference for the contrast model and positive values indicate preference for the hurdle model. **F** Proportion of absent responses in the detection task (circles with solid lines; cf. Supplementary Fig. S3A) compared to the probability of zero reproduction (i.e., not passing the hurdle) in the reproduction task (triangles with dashed lines) as predicted by the Hurdle-Gaussian model. **G** Linear regression between the observed effect (biased condition minus control condition) on the proportion of absent responses (logit-transformed) in the detection task and the model-predicted effect (biased condition minus control condition) on the probability of not passing the hurdle (logit-transformed) in the reproduction task. This analysis directly tests the relation between the proportion of absent responses in the detection task, and the model-predicted (Hurdle-Gaussian model) proportion of zero reproductions. The gray shaded area around the regression line corresponds to the 95% confidence interval.

As in Experiment 1, we asked participants to detect dim Gabor patches (detection task) or on a random subset of trials to reproduce their subjective experience of the patches to the best of their ability. To evaluate the effect of the manipulations on the detection task, we followed the same procedure and analysis pipeline described in Experiment 1. The qualitative and statistical pattern of results was identical to the results we described for Experiment 1, as can be seen in Figs. 5A through 5G (for a more detailed verbal description of the results of Experiment 2, see Supplementary Note S3). These results confirm that when comparing the contrast and hurdle models, the attentional cue effect is best explained by the contrast model, while the base-rate and payoff effects are best explained by the hurdle model. These results also confirm that the proportion of zero-contrast responses in the reproduction task is well-predicted by a bias effect in the detection task, substantiating the claim that the proportion of *non-zero* reproductions is caused by a decision criterion shift in the detection task.

### Effects on reproduced contrast

The Hurdle-Gaussian model allowed us to independently estimate the effect our contextual manipulations had on the reproduction hurdle and reproduced contrast, and their overall contribution to the reproduction measure. This modeling approach was able to recover the effect of each manipulation on the overall reproduced contrast responses (*zero* and *non-zero* contrast; see Figs. 4A and 5C), and also allowed us to directly relate the effect of the hurdle parameter on proportion of *zero* reproductions to the decision threshold in the detection task (see Figs. 4D, E and 5F, G). Together, these results show that the generative model of the observers during the reproduction task is best characterized as a two-step process that consists of a binary decision (as reflected in the reproduction hurdle) and a linear (reproduced contrast) component (see Hurdle + Contrast model, right panel of Fig. 3A).

**Fig. 6:**
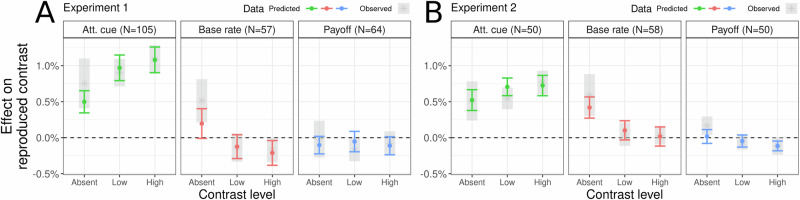
Hurdle-Gaussian modeling of reproduced contrast in Experiment 1 and 2. Observed and model-predicted effect on reproduced contrast in Experiment 1 and 2. The effect on reproduced contrast (only non-zero reproductions) in Experiment 1 (**A**) and Experiment 2 (**B**) is calculated as the difference between the average non-zero reproduction of the bias and control condition independently for each contrast level. Average observed data is plotted as a gray asterisk, whereas the effect on reproduced contrast predicted by the full Hurdle-Gaussian model is plotted as a dot in green (attentional cue), red (base rate) or blue (payoff). Gray bars and colored error bars indicate the 95% confidence interval.

Having separately modeled the effect of the reproduction hurdle, we now turn to the effects on reproduced contrast predicted by the Hurdle-Gaussian model. To assess the effect on reproduced contrast, we calculated the difference in *non-zero* reproduced contrast between the biased and control conditions for each bias manipulation and contrast level, both in the observed data and the model-predicted data of Experiment 1 and 2 (see Fig. 6). The model-predicted effects are, for the most part, the same as the observed effects. We see a consistent positive effect of the attentional cue in all contrast levels across both experiments. In contrast, the base rate and payoff condition exert little to no effect on reproduced contrast, except maybe for absent trials in the base rate condition. Additionally, in absent trials of the attentional cue (Experiment 1) and absent trials of the base rate condition (both experiments), the model-predicted effects are smaller than the observed effects. This reduction in the size of the effects is plausibly the result of removing the effect of the reproduction hurdle from the effect on reproduced contrast. To summarize, once we account for the effect on the reproduction hurdle, only the attentional cue consistently shows an effect on reproduced contrast, whereas the base rate and payoff manipulations show little effect on reproduced contrast, except for a small effect on absent trials in the base rate condition.

### Experiment 3: reproduction without detection

Somewhat counterintuitively, these results suggest that the proportion of zero-contrast reproductions in the payoff and base rate conditions can change, even if the average experienced contrast in those conditions stays the same. If true, this would mean that attentional cues affect what is consciously perceived (experienced contrast), whereas base rate and payoff merely affect the reproduction hurdle for *non-zero* reproductions. This interpretation hinges on the assumption that non-perceptual biases affect whether a *non-zero* reproduction response is given. Alternatively, one might argue that the changes in the ratio of *zero* to *non-zero* reproductions observed in the base rate and payoff conditions also reflect changes in the subjective experience of the observer, such that higher stimulus prevalence or higher cost for misses causes the stimuli to be consciously perceived more often. This debate is in fact the one we started with–is a manipulation perceptual, or not? Based on the striking similarity and association between the criterion shifts in the detection task and the hurdle in the reproduction task (see Figs. 4D, E, and 5F, G), we considered that one route towards resolving this issue is to ask whether the biases in the reproduction hurdle may have been artificially induced by the requirement to also respond to a concurrent (and unpredictable) detection task. If this is the case, then we should be able to isolate a “pure” effect of our manipulations on reproduction by removing the concurrent detection task.

To decide between these two accounts, we conducted a control experiment (Experiment 3) in which we kept all physical stimulation the same, but removed the detection task and the associated instructions that emphasize the presence or absence of targets. Instead, participants were asked to simply reproduce the contrast they perceived, while we kept the bias manipulations in place and the trial layout the same as in Experiment 2. Because the payoff manipulation is contingent on detection responses (punishing misses more strongly than false alarms), only the attentional cue and base rate manipulations could be deployed in this experiment.

**Fig. 7:**
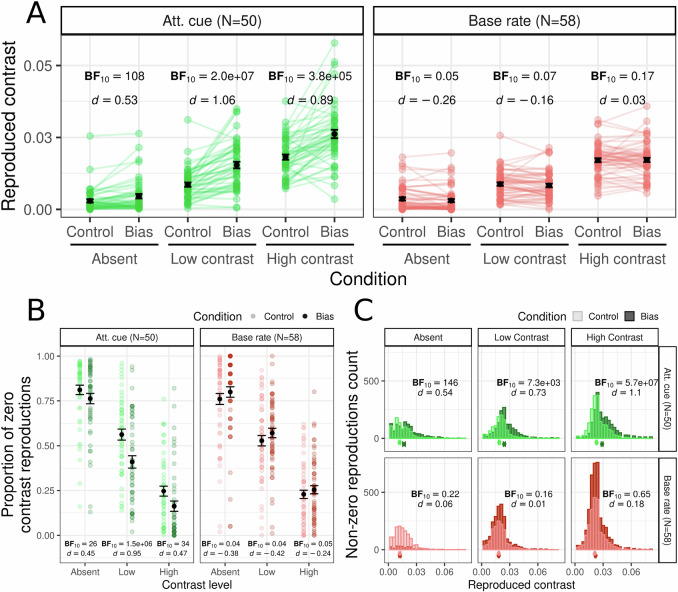
Experiment 3. Reproduction task results. **A** The average reproduction (both zero and non-zero contrast reproductions) of each participant along with the group average for each bias source and condition. **B** Average proportion of zero reproductions (zero contrast reproduction; “absent” reproduction) per participant and condition. **C** Distribution of non-zero reproductions (higher-than-zero contrast reproduction; “present” reproduction). All the reproduction responses of all participants are plotted as a histogram. The average reproduced contrast for each histogram is plotted as a dot below the distribution. Note that 11 participants in the base rate condition could not be plotted because they did not have any non-zero reproduction on absent trials. All error bars indicate the SEM. BF values correspond to a paired one-sided t-test with a Cauchy prior (centered around zero with a width of 0.707), d values indicate Cohen’s d effect size.

When taking into account all reproduction responses (both *zero* and *non-zero* contrast), there was decisive evidence (BF_10_ > 100, *d* > 0.5) for an effect across all contrast levels in the attentional cue, whereas in the base rate condition, there was moderate to strong evidence for no effect across all contrast levels (BF_10_ ≤ 0.17, *d* ≤ 0.26; see Fig. 7A). Next, we again calculated the proportion of *zero* contrast reproductions and the average *non-zero* reproduced contrast to assess the effects of the manipulations on observers’ reproduction hurdle and reproduced contrast. In the attentional cue condition, cued trials resulted in a lower proportion of *zero* reproductions across all contrast levels (BF_10_ ≥ 26, *d* ≥ 0.45). Conversely, however, there was strong evidence for no effect across contrast levels in the base rate condition (BF_10_ ≤ 0.042, *d* ≤ 0.42; see Fig. 7B). When assessing the average *non-zero* reproduced contrast, there was decisive evidence for an effect in the attentional cue across all contrast levels (BF_10_ > 100, *d* ≥ 0.54), whereas in the base rate condition, there was moderate evidence for no effect in absent and low contrast trials (BF_10_ ≤ 0.22, *d* ≤ 0.06), and anecdotal evidence for no effect in high contrast trials (BF_10_ = 0.65, *d* = 0.18; see Fig. 7C).

Crucially, the effect of base rate on the proportion of *zero* reproductions in Experiments 1 and 2 disappeared once the detection task was removed. These results confirm that the decision criterion adopted in the detection task in Experiments 1 and 2 had contaminated reproduction responses, such that it shifted the proportion of *zero* reproductions without affecting conscious perception. In contrast, the effect of the attentional cue on the proportion of *zero* reproductions and on the *non-zero* reproduced contrast was not affected by the removal of the detection task. The results confirm the interpretation that attentional cues influence subjectively perceived contrast (conscious perception), while the effect of base rate is decisional in nature.

## Discussion

Using a Hurdle-Gaussian model^30^, we were able to separate the contribution of the decision criterion from observers’ reproduced contrast in the reproduction task. Our approach shows that on the surface, all manipulations influenced the decision criterion (i.e., increasing the number of present responses on all contrast levels). However, we show that this result is driven by distinct effects on the generative model of the observer, which we interpret as partially perceptual and partially decisional. The base rate and payoff manipulations were predominantly driven by a decision criterion shift, while the attentional cue manipulation selectively caused a shift in reproduced contrast. We validated our modeling approach by replicating the results of Experiment 1 in a new sample (Experiment 2). Further, we show that the effect of base rate on zero contrast reproductions (i.e., the hurdle effect) was caused by the presence of a detection task in Experiment 1 and 2. When removing the detection task in Experiment 3, the effect on zero reproductions disappeared, despite the large difference in the proportion of target absent presentations between the control and bias condition. This finding rules out a potential perceptual explanation for the threshold effect in reproduction by comparing the effect of the attentional cue to the effect of the base rate manipulation after removal of the detection task (Experiment 3).

Decision criterion contamination is a prevalent issue in the study of conscious perception, but also more broadly in psychophysics, perceptual decision-making and metacognition. Although plenty of work has been devoted to this topic^5,34–39^, little work has been done to investigate which contextual influences have perceptual and which have non-perceptual effects during conscious detection. Without this work, it is hard to assess the degree to which the consciousness literature is confounded by non-perceptual influences. Here we fill this gap by explicitly manipulating the criterion and showing that typical contextual influences like induced statistical regularities (base rate) and task utility (payoff) have non-perceptual effects. Such influences are likely to confound experimental outcomes as they occur naturally across experiments (i.e., differences in the ratio of target to non-targets, differences in the perceived utility of certain stimulus-response combinations and so forth). Exogenous attentional cues, on the other hand, alter what is consciously experienced.

Indeed, previous research has long claimed that covert exogenous cues alter perceived contrast, without altering the response criterion^28,40–42^ (see ref. ^43^ for a review). In support of this position, neurophysiological and neuroimaging data have shown that the effect evoked by exogenous cues is similar to the effect of directly varying stimulus contrast (see ref. ^44^ for a review). Our results extend this line of research by directly assessing the effect of covert exogenous attention over perceived contrast using an independent behavioral benchmark to assay changes in subjective experience. We show across three experiments that exogenously cued stimuli are more readily reported but also reported as having a higher contrast than non-cued stimuli. Note that this effect may not generalize to other attentional effects, as other studies have shown that the allocation of attention may also influence report without affecting perceptual processing^45,46^.

Despite this converging evidence, an alternative account argues that the effects of exogenous cues on appearance may be explained by low-level sensory interactions between the luminance polarity of the cue and target stimulus^47^, but see ^48^ or by decisional biases^49,50^, but see ^51–53^, rather than changes in perceived contrast. For instance, it has been argued that comparative judgments of the type used in research suggesting exogenous cues influence perceived contrast e.g^28^^.^ may bias participants to report the cued item as having higher contrast, even if the cue did not influence perceived contrast. While these claims have been addressed in various works (see ref. ^43^ for a review), it is important to note that our study does not employ any of the tasks previously criticized as leading to decisional biases. Instead, by combining a detection and reproduction measure, we provide converging evidence that exogenous cues influence subjective appearance, while also separating such perceptual effects from decisional biases.

While past research has shown that one can disentangle perceptual from non-perceptual effects using discrimination tasks^1,2,54,55^, the cornerstone of consciousness research is the assessment of awareness using present/absent detection tasks^56–58^. Here, we show that reproduction measures, when intermixed with a detection task, are also prone to the pervasive influence of decisional biases. These results differ from our previous work in which reproduction uniquely captured the perceptual component in a decision process. Strikingly, the mere presence of a detection task is enough to affect the ratio of *zero-* to *non-zero* reproductions. In contrast, the removal of the detection task (Experiment 3) abolishes this effect. These findings reveal that the act of detection itself can introduce non-perceptual biases into measures of conscious experience, even when using an appearance-based metric like reproduction as a safeguard.

The question remains as to how the presence of detection requirements influences subsequent reproduction responses. Based on our previous work^1,2^, we argue that the reproduction task allows participants to report the strength of their perceptual experience directly, without invoking a decision criterion that can lead to bias. However, in the present study, we show that intermixing the reproduction task with a detection task causes participants to make a detection judgment on every trial, including those in which they were asked to reproduce. Thus, when reproduction trials are intermixed with detection trials, the generative process of reproduction responses is better captured by the Hurdle + Contrast model, where a criterion-based decision precedes the reproduction response. In contrast, in Experiment 3, where the detection task was removed, responses are more consistent with the Contrast-only model. Apparently, the requirement to detect introduces a hurdle (threshold) that sensory experience has to cross to determine whether to reproduce on reproduction trials. In a reproduction-without-detection task, the Hurdle does not play a role so that the reproduction response itself provides a more direct assessment of conscious experience.

Although traditional reproduction (appearance-based) tasks often rely on presenting a target in all trials, here we show that this setup can be extended to assess conscious perception by including both target present and target absent trials. In such a paradigm, reproduction-without-detection reflects the subjective strength of the perceptual experience: While *non-zero* reproductions indicate trials in which the observer had some perceptual experience of the stimulus, *zero* reproductions reflect the absence of any perceptual experience. The reproduction-without-detection framework enables the direct comparison of neural or indirect behavioral signatures of trials with conscious perception (*non-zero* reproductions) and those without (*zero* reproductions). Furthermore, the reproduction task can be further combined with techniques used to manipulate awareness, such as masking, the attentional blink, or continuous flash suppression (CFS), to contrast the underlying processes of *non-zero* reproductions (consciously perceived stimuli) against *zero* reproductions (stimuli that were not consciously perceived). This potentially even allows one to compute a reproduction d’ based on these responses, for example taking the proportion of non-zero reproductions on target present trials as the hit rate and the proportion of non-zero reproductions on target absent trials as the false alarm rate. Further, because reproduction responses provide a continuous measure of subjective experience, they also support more nuanced analyses in which the graded strength of subjective experience can be related to trial-level neural measures (e.g., EEG). In this way, the reproduction approach offers an alternative methodology for consciousness research, providing a continuous, criterion-free index of subjective experience. As a cautionary note: obtaining reproduction metrics is not always straightforward. For example, the item to be reproduced must have some scalable quality like contrast, color, length or orientation to perform the reproduction on. Further, certain manipulations like masking or CFS might make it difficult to properly create a reproduction item (i.e., the stimulus participants adjust to match what they saw) that has the same perceptual characteristics as the original target.

Thus, despite the advantages of the reproduction approach, it may not always be possible to implement a reproduction task. Below we consider a number of alternative approaches that have been proposed to measure subjective experience, each with its own advantages and limitations.

A widely used approach leverages metacognitive assessments in which observers rate their confidence about their perceptual judgments. A large body of research shows that confidence tracks perceptual performance reasonably well^59–63^, and confidence has often been interpreted as a proxy for perceptual strength^64–67^. Some have also suggested that confidence can help distinguish perceptual from decisional influences^68^. However, in recent work^2^, we found that confidence was not only sensitive to perceptual manipulations but also shifted under decisional manipulations. This and other work^69,70^ shows that confidence reports incorporate decisional biases. One way to address this limitation is to use aggregated measures of metacognition, such as M-ratio. M-ratio captures how well confidence discriminates between correct and incorrect responses while controlling for first-order sensitivity and bias^62,71^. However, there is currently little evidence that this approach can dissociate perceptual from non-perceptual effects. Exploratory analyses^2^ suggest that metacognitive efficiency is also affected by decisional manipulations, such as base rate.

Another popular method is the perceptual awareness scale (PAS), which provides a four-point rating of subjective visual experience ranging from no experience to absolutely clear perception^72^. The PAS was designed as a graded scale of perceptual awareness. Some have used the “No experience - No Impression of the stimulus” (or similar phrasing) designation on that scale to isolate “unconscious” trials. However, PAS responses still require participants to set decision criteria when distinguishing between different levels of experience. Recent work demonstrates^6^ that such criteria can be shifted by non-perceptual influences, showing that the PAS is not immune to decisional contamination.

Finally, unlike yes/no detection tasks (like the 1AFC detection task used in Experiments 1 and 2), two-alternative (2AFC) and two-interval forced-choice (2IFC) tasks are often considered criterion-free, since participants must choose between two simultaneously or sequentially presented stimuli^15,28,29^. Building on this framework^73^, a two-interval confidence task was introduced in which observers perform two orientation discriminations and then indicate which interval they felt more confident about (for a detailed critique see ref. ^74^). This approach seeks to reduce reliance on absolute criteria by anchoring judgments to relative differences across intervals. While elegant, the relationship of this approach to other detection-based measures remains debated^74,75^.

In summary, we show that reproduction-without-detection provides a useful alternative for assaying experienced stimulus strength in paradigms that contain target absent trials, which would otherwise rely on explicit detection reports. Specifically, we find that including a detection task in the experimental block contaminates reproduction responses with non‑perceptual biases. Comparing Experiments 1 and 2, in which the detection and reproduction task were randomly intermixed across trials, with Experiment 3, in which the detection task was removed but physical stimulation and contextual manipulations were kept identical, revealed that the mere presence of a detection task - that is, the requirement to occasionally make present/absent judgments - can alter the balance between zero and non-zero reproductions, and thereby introduce non-perceptual biases into reproduction responses. This underscores the importance of exercising caution when employing and interpreting detection-based paradigms in consciousness research. When the main concern is contamination by criterion shifts, reproduction-without-detection may be better suited than yes/no detection to index conscious experience. This does not mean that reproduction is bias‑free or exclusively perceptual. As we discuss and show, reproduction can also be influenced by a variety of post‑perceptual factors. Our contribution is to show that detection requirements are themselves a powerful source of non‑perceptual bias that can easily spill over into other measures of conscious experience, such as reproduction.

## Methods

This study consists of three experiments. Methods and analyses are the same for all three experiments unless stated otherwise.

### Participants

All experimental procedures (Experiments 1, 2 and 3) were approved by the Ethics Review Board of University of Amsterdam. Informed consent was obtained in accordance with the approved procedures. 251 participants took part in Experiment 1, 168 in Experiment 2, and 109 in Experiment 3. Out of these participants, 238 (187 women, 46 male and 5 other; average age 20.8, SD = 2.98), 158 (131 women, 24 male and 3 other; average age 21, SD = 2.9) and 109 (94 women, 13 men and 2 other; average age 19.9, SD = 2.25) participants were analysed, respectively (see Supplementary Note S4 for participant removal criteria; self-reported gender). Participants were recruited through the lab pool of the University of Amsterdam and the Free University Amsterdam, and received a base monetary compensation or research credits. Additionally, participants could earn an extra amount of research credits or money depending on their performance in Experiments 1 and 2. The extra reward maximally consisted of a third of the base payment. On average the total payment was similar across conditions. Each participant completed one session of roughly 90 (Experiment 1 and 2) or 60 minutes (Experiment 3), including instructions, practice and breaks.

Participants were outliers if their staircase thresholds, signal detection theory d’ or reproduction error fell outside four standard deviations from the sample mean across all conditions (attentional cue, base rate and payoff) but independently for each experiment. Participants with a signal detection theory d’ below zero were also removed. In total 12 participants were removed in Experiment 1, 10 participants in Experiment 2, and 1 participant in Experimentent 3 (see Supplementary Note S4 for the detailed number of outliers removed per condition).

After filtering, the data of 105 (attentional cue), 57 (base rate) and 64 (payoff) participants was analysed in Experiment 1. As a result of the optional stopping rule we applied in the data collection of Experiment 1 the participant count in the attentional cue was considerably higher compared to the base rate and payoff condition. However, note that we replicate the results of Experiment 1 in Experiment 2 (where the participant count is relatively even across conditions). In Experiment 2, the data of 50 (attentional cue), 58 (base rate) and 50 (payoff) participants was analysed after filtering. In Experiment 3, the data of 50 (attentional cue) and 58 (base rate) participants was analysed after filtering.

### Sample size

The sample size of Experiment 1 was determined by applying an optional stopping rule that depended on the main comparisons of interest, whereas for Experiment 2 we conducted a sequential sampling plan based on the effect sizes of Experiment 1. In Experiment 1 we collected the data of 30 participants, removed outliers and ran a Bayesian t-test between the control and biased condition in the detection (rate of ‘present’ responses) and reproduction task (reproduced contrast). These tests follow the same procedure as the main hypothesis tests as reported in Fig. 1C, D. For each comparison, if there was at least moderate evidence for the presence or absence of an effect (for either the null or the alternative hypothesis; BF_10_ > 3 or BF_10_ < 0.33) in both tasks we stopped data collection, otherwise we collected five more subjects and repeated the process. In this framework optional stopping or data peaking is not considered problematic^76^. In Experiment 2, we aimed to collect a sample that allowed us to detect at least the smallest effect size found in Experiment 1 (Cohen’s *d* = 0.4) with at least moderate evidence (BF_10_ > = 3 or BF_10_ ≤ 0.33). We used the BFDA R package^77^ for Bayesian Design Analysis^78^. A sequential sampling plan indicated that a sample of 50, 65 and 85 participants would yield respectively a power of 70%, 80% and 90% to detect the aforementioned effect. We decided to collect the data of at least 50 participants and continue testing until all comparisons yielded moderate evidence for the null or alternative hypothesis (BF_10_ > = 3 or BF_10_ ≤ 0.33), or until we collected 85 participants (50 and 85 refer to the participant count after filtering outliers). In Experiment 3 we aimed to collect the same number of participants as in Experiment 2 after removing outliers.

### Tasks and trial layout

Participants had to detect (detection task) or to reproduce (reproduction task) a dim Gabor patch (see Stimuli section below), also referred to as the target patch. Each trial started with a 1000 ms fixation period (during the first 500 ms the central dot of the fixation turned white to indicate the start of the trial), followed a target period of 25 ms, followed by a second fixation period (600, 700 or 800 ms in Experiment 1, or fixed at 600 ms in Experiment 2 and 3), after which participants were prompted to either make a detection response or to reproduce the target patch (until response; see Fig. 1). Crucially, in Experiments 1 and 2, participants did not know which task they had to perform until after stimulus offset. In Experiment 3, the trial layout was identical to the one in Experiment 2, but participants only performed the reproduction task.

On detection trials, the text *Absent - Present* appeared centered on the screen until participants answered by pressing the left (to answer “absent”) or right (to answer “present”) button of the mouse. On reproduction trials, the word *Reproduce* (in Experiment 1) or the letter *R* (in Experiments 2 and 3) appeared centered on the screen until participants used the wheel of the mouse to adjust the contrast of a new Gabor patch, from here on referred to as the reproduction patch. After scrolling up or down for the first time, the word *Reproduce* or the letter *R* disappeared to make space for the reproduction patch, initially presented at zero contrast. The reproduction patch was presented intermittently at a constant rate of 1 Hz with the same presentation time as the target patch. When adjusting the contrast of the reproduction task, each step increased the contrast of the patch by a factor of 1.0869, or it decreased by a factor of 0.92, meaning that scrolling up one step and then one step down would bring the contrast back to its original value, that is, before the first step up. The minimum *non-zero* contrast value the patch could have was 0.07% (theoretical minimum contrast of the monitor). The patch contrast went from 0 to 0.07% in one scroll step and vice versa from 0.07% to 0. In Experiment 1, the contrast of the reproduction patch was increased by scrolling up and decreased by scrolling down. In Experiments 2 and 3, the scrolling direction was counterbalanced within a block, such that in some trials scrolling up increased the contrast of the patch, whereas in the rest of the trials scrolling up decreased the contrast of the patch. Participants were informed in each trial about the scrolling direction scheme applied in that trial (see “Methods” *- General procedure* for a detailed account and rationale).

### Stimuli

All three experiments were scripted and ran in behavioral cubicles using PsychoPy^79^ and Python^80^. Stimuli were presented on a monitor with a resolution of 1920 × 1080 and a 120 Hz refresh rate. The width of the monitor was 58.4 cm placed at a viewing distance of 75 cm (Experiment 1 and base rate condition of Experiment 2) or 52.6 cm placed at a viewing distance of 80 cm (attentional cue and payoff of Experiment 2 and all conditions of Experiment 3). Stimuli consisted of Gabor patches spanning 2.5° of visual angle in Experiment 1, and 2° in Experiments 2 and 3, with a spatial frequency of 2 cycles per degree (cpd), a negative phase of 1 and an orientation of 45° or 125°. In present trials the target patch was presented either on the left or right side of the fixation (3° off-center across the x-axis and centered across the y-axis). The target patch orientation and position were counterbalanced across present trials. In Experiments 1 and 2, the contrast of the target patch was titrated for each participant to create three contrast levels: absent, low and high (see the *Staircase procedure* in the next section for a detailed account). In Experiment 3, we used the average low and high contrast values obtained from Experiments 1 and 2. These three contrast levels allowed us to evaluate whether participants could perform the reproduction task–as they should result in three reproduced contrast levels. The reproduction patch was identical to the target patch (presentation duration, size, spatial frequency, phase and orientation) except that it was presented at the center of the screen, and its contrast was adjusted by the participant while it (re-)appeared with a frequency of 1 Hz. The attentional cue consisted of a dim gray (RGB(105, 105, 105)) circle (0.2°) presented for 66.67 ms before the target patch (SOA 116.67 ms) either on the left or on the right of the fixation, 3° off-center across the x-axis (that is, aligned with target patch position across the x-axis) and 1.2° off-center across the y-axis in Experiment 1 or 1.5° in Experiments 2 and 3. This setup is largely identical to the way cues were presented in ref. ^28^.

### Staircase procedure

To effectively create two contrast levels on present trials, the contrast of the target patches was adjusted for each participant using two staircase procedures, one aimed at a 33% hit-rate (*low contrast*), and one aimed at a 66% hit-rate (*high contrast*). Note, however, that *high contrast* patches had a low contrast in absolute terms and were comparatively high relative to *low contrast* patches but were still very difficult to see. Both staircases had an initial value of 0.03 (Michelson contrast) and were updated only in stimulus present trials. Each staircase was updated on a trial-by-trial basis with a different weight for correct and incorrect responses using a ratio of 2:1 in the low contrast staircase, and 3:1 in the high contrast staircase. For example, after a mistake, the low contrast staircase was updated by increasing the contrast of the target patch by two steps, whereas the contrast was decreased by one step after a correct response. To account for the non-linear character of contrast perception^81^, the step size of both staircases was calculated by transforming the current target patch contrast to a linear perceived space (by rising it to the power of 0.7) and then dividing it by 15 (*step size* = *current contrast* ^0.7^ / 15). This made the step size smaller when the target contrast was low or bigger when the target patch contrast was high, effectively accounting for the non-linearity of contrast perception. Then the step size was added or subtracted to the target patch contrast and transformed back to a non-linear contrast scale by raising it to the power of *1/0.7*.

### Bias manipulations

To prevent order effects, we employed 3 between-participants manipulations (attentional cue, base rate and payoff) to bias participants towards “present” responses (bias-present condition), each paired with a within-participant control condition. In Experiment 3, we employed only the attentional cue and base rate condition (both accompanied by a within-participant control condition). See Fig. 1B for a graphical depiction of the three bias manipulations. Trials in the three bias conditions were identical except for the following details: (i) In the attentional cue condition, a lateralized cue was presented on every trial, shortly before the onset of the target period. The cue was always presented and did not predict the absence or presence of the target stimulus. However, it did predict the position of the target on present trials because it always occurred on the same side of the target. This manipulation is highly similar to the manipulation in ref. ^28^, which has been shown to produce changes in perceived contrast. (ii) In the base rate condition, there were nine times more present trials than absent trials, while in all other conditions the ratio between present and absent trials was even. In all conditions, present trials were composed of 50% low contrast trials and 50% high contrast trials. (iii) In the payoff condition participants were differentially punished for incorrect detection responses, so that misses costed them five times more than false alarms. The asymmetrical payoff was applied exclusively to the detection task, whereas in the reproduction task there was a flat penalty for any incorrect response (Experiment 1) or there was no penalty (Experiments 2 and 3). Note again that in Experiments 1 and 2, participants did not know whether they had to perform the detection or the reproduction task until the end of each trial^similar to^ ^1^. In Experiment 3 participants only had to perform the reproduction task. Each of the bias manipulations had an accompanying control condition, each with a flat penalty for any incorrect response in the detection task, an equal proportion of present and absent trials, and no cue preceding the target patch.

### General procedure

For each task (detection and reproduction), participants received instructions and completed extensive practice (see Supplementary Note S5 for a detailed description of the procedure). Next, participants completed two blocks, one for each condition (control and bias), with 200 trials per task in random order (detection and reproduction). Summing up to 800 trials in Experiments 1 and 2, and 400 trials in Experiment 3. The experiment was further divided into 50-trials blocks. Each block in Experiments 1 and 2 started with a message that reminded participants about the relative frequency of absent and present trials (base rate condition), about the cost for misses and false alarms (payoff condition), or were reminded that the cue did not predict the presence of the target (attentional cue condition). In Experiment 3, blocks started with a message indicating that a new block was about to start. In Experiments 1 and 2, participants received feedback on their block-level performance at the end of each block (see Supplementary Note S6 for a detailed description of the feedback and Supplementary Note S7 and S8 for an example). In Experiment 3, participants did not receive any feedback and were merely informed about the percentage of the experiment they had completed at the end of each block. Participants had the opportunity to have a short break if desired before starting a new block. Additionally, there was a mandatory 3-minute break every 200 trials.

### Differences between Experiment 1, 2 and 3

Experiment 2 was almost identical to Experiment 1, except for the following changes. In Experiment 1, participants were asked to match their subjective experience of the target patch by adjusting the contrast of the reproduction patch. However, during the instructions of the reproduction task, participants received feedback that may have overemphasized the mismatch between their experience and the objective contrast of the target patch, for example, when a dim patch was presented, but the reproduction patch was made fully invisible. Furthermore, reproductions that were too far off decreased participants’ extra reward, and although participants were not informed about the direction of their mistakes in the reproduction task – thus preventing them from using the feedback to correct their responses – this overall emphasis on the objective correctness of their reproductions may have pushed participants to optimize for correctness rather than their subjective experience. To address these issues, we modified the instructions of the reproduction task in Experiment 2 to emphasize that the reproduction patch should match their subjective experience of the stimulus, rather than the objective contrast of the target patch. Additionally, participants no longer receive feedback on the number of accurate reproductions in Experiment 2, and the accuracy of their reproductions did not count towards their extra reward.

Another issue addressed in Experiment 2 was the way in which participants adjusted the contrast of the reproduction patch. In Experiment 1, the scrolling direction that increased or decreased the contrast of the reproduction patch was fixed across the entire experiment. This could have resulted in a response bias due to motoric habituation that may have unequally affected the manipulations given the asymmetrical distribution of present trials across conditions. To overcome this problem, the scrolling direction of the mouse wheel was randomized across trials and counterbalanced within each block, meaning that in some trials scrolling up increased the contrast of the reproduction patch, whereas in other trials scrolling up decreased the contrast of the reproduction patch. Participants were informed about the scrolling direction; on each trial a label appeared above and below the reproduction patch to indicate which direction increased or decreased the contrast of the patch (‘increase visibility’ / ‘decrease visibility’). To make the labels less intrusive during the task, they turned dimmer once the participant started adjusting the contrast of the reproduction patch (see Supplementary Fig. S2 for an example of the reproduction prompt).

A third issue we addressed in Experiment 2 was the removal of the variable fixation period in prior to the response. In Experiment 1 this was a randomly chosen variable interval of 600, 700 or 800 ms, which was fixed at 600 ms in Experiment 2 and 3.

Finally, even though participants did not know which task they had to perform on each trial in Experiments 1 and 2, we wanted to evaluate whether the detection task influenced reproduction responses by covertly preparing participants to give a present/absent response (detection decision) even on reproduction trials. If true, this might have caused absent decisions to express themselves as zero contrast reproductions, even when participants experienced *non-zero* contrast. To gauge participants’ true experience of the stimulus without the potentially confounding influence of the detection task on the reproduction response, we conducted a control experiment (Experiment 3) in which we kept all physical stimulation identical, including base rate and attentional cue manipulations, while selectively removing the detection task and its associated instructions regarding stimulus presence or absence. Instead, participants were told that the Gabor patches would be more visible in some trials and very hard to see, or invisible, in other trials. We reasoned that if the detection task had not influenced how participants behaved in the reproduction task, the pattern of results should not be affected when removing it. However, if the detection task inadvertently affected how participants respond in the reproduction task, the reproduction task without detection would be expected to show a different pattern of results compared to Experiments 1 and 2, despite identical physical stimulation. Specifically, we would expect the effect on the proportion of *zero* reproductions to disappear in Experiment 3, if it was artificially induced by the detection requirements present in Experiments 1 and 2.

### Reporting summary

Further information on research design is available in the Nature Portfolio Reporting Summary linked to this article.

## Supplementary information

Supplementary Information

Reporting Summary

Transparent Peer Review file

## Data Availability

All the data (raw and processed) generated in this study^82^ has been deposited at https://osf.io/tbfne/ (DOI: 10.17605/OSF.IO/TBFNE).

## Code availability

All the scripts used in the analysis as well as the code to generate the plots are publicly available at https://osf.io/tbfne/ (DOI: 10.17605/OSF.IO/TBFNE).
