# Supplementary material for: The act of detecting a stimulus contaminates measures of conscious experience with decision biases
Source: Nat Commun. 2026 May 8;17:6254. doi: 10.1038/s41467-026-72567-6 (PMC13376516; doi:10.1038/s41467-026-72567-6)
Supplement: Supplementary file 1 — Supplementary Information [file 41467_2026_72567_MOESM1_ESM.pdf]

The act of detecting a stimulus contaminates measures of conscious experience with decision biases

Nicolás Sánchez-Fuenzalida[1,2,3,4,✉], Chris Jungerius[3,5], Stephen M. Fleming[6,7], Simon van Gaal[1,2], and Johannes J. Fahrenfort[1,2,3,4]

[1] Department of Psychology, University of Amsterdam
[2] Amsterdam Brain & Cognition, University of Amsterdam
[3] Department of Applied and Experimental Psychology, Free University Amsterdam
[4] Institute for Brain and Behavior Amsterdam, Free University Amsterdam
[5] Swammerdam Institute for Life Sciences, University of Amsterdam
[6] Institute of Cognitive Neuroscience and Department of Experimental Psychology, University College London
[7] Max Planck-UCL Centre for Computational Psychiatry and Ageing Research, University College London

✉ Correspondence: Nicolás Sánchez-Fuenzalida <nicolas.carvajalsanchez@unibas.ch>

Supplementary Information

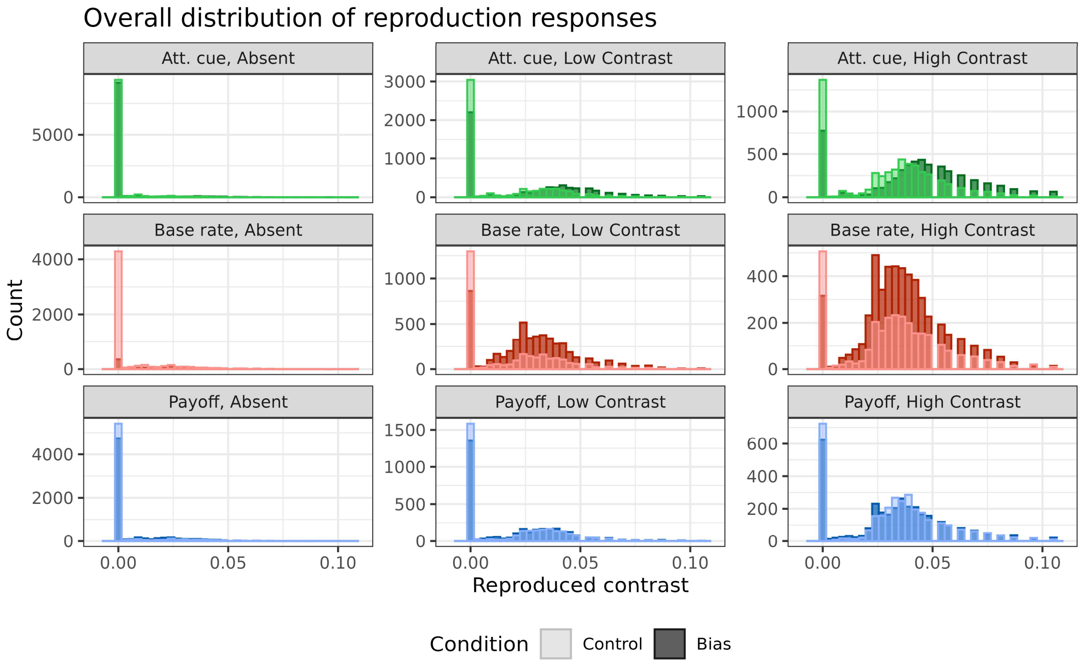

Figure S1: ***Experiment 1 distribution of all reproduction responses****. All the reproduction responses of all participants are plotted as a histogram.*

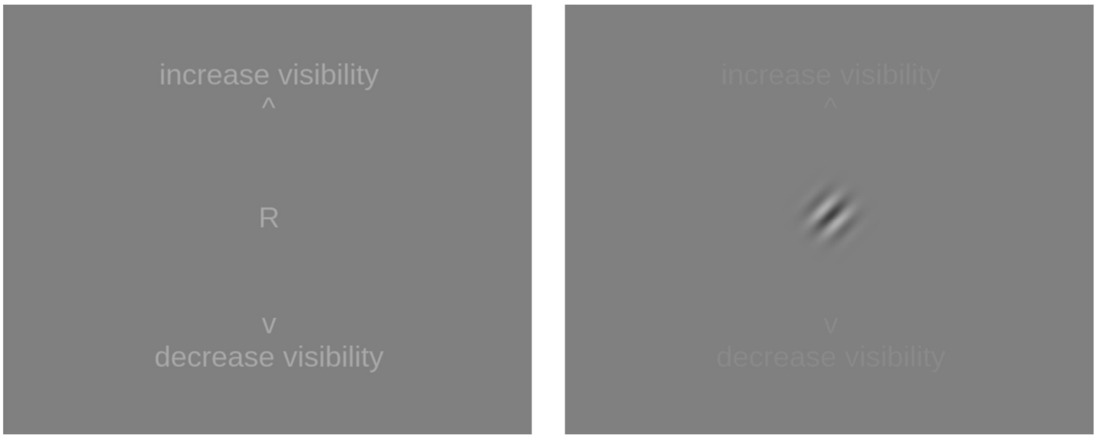

Figure S2: ***Experiment 2: Reproduction task prompt.*** *On each reproduction trial participants had to adjust the contrast of the reproduction patch using the wheel of mouse. The scrolling direction was counterbalanced and randomly presented within a block. The prompt to reproduce also indicated the scrolling direction associated with an increase or decrease in contrast (left panel). After scrolling once, the R disappeared, and the scrolling direction aid was made almost translucent to make it less intrusive (right panel).*

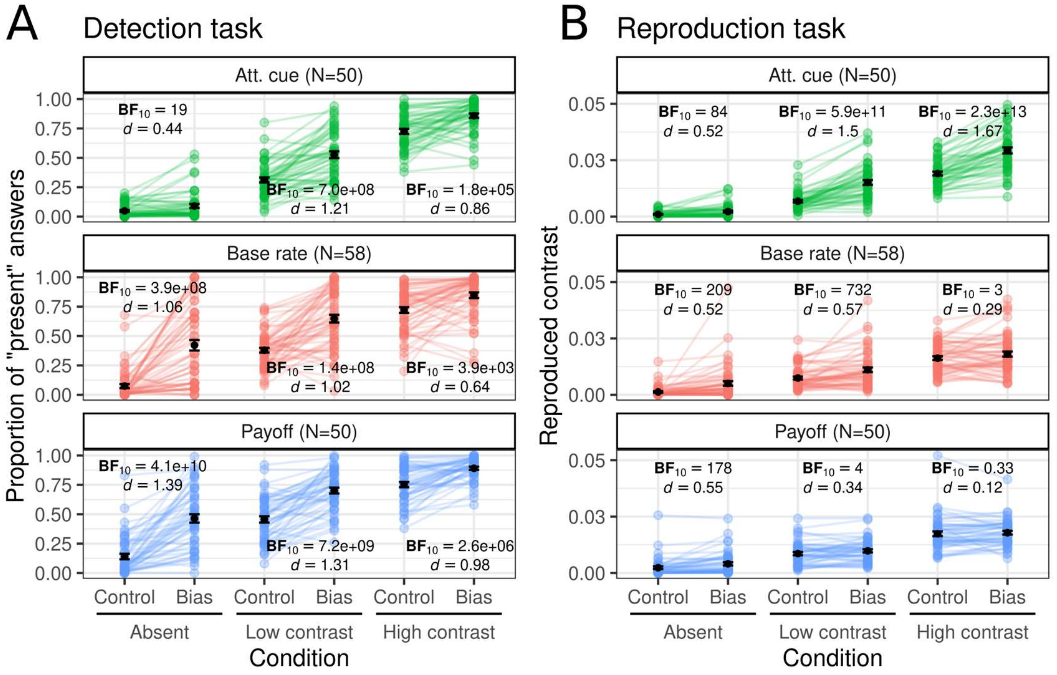

Figure S3: ***Experiment 2 detection and reproduction results****. A)* ***Detection task.*** *The proportion of 'present' responses of each participant along with the group average for each bias source and condition. B)* ***Reproduction task task.*** *The average reproduction of each participant along with the group average for each bias source and condition. The average of each condition is depicted as a black dot. All error bars indicate the SEM. All BF values correspond to a paired one-sided t-test with a Cauchy prior of 0.707. d values indicate Cohen's d effect size.*

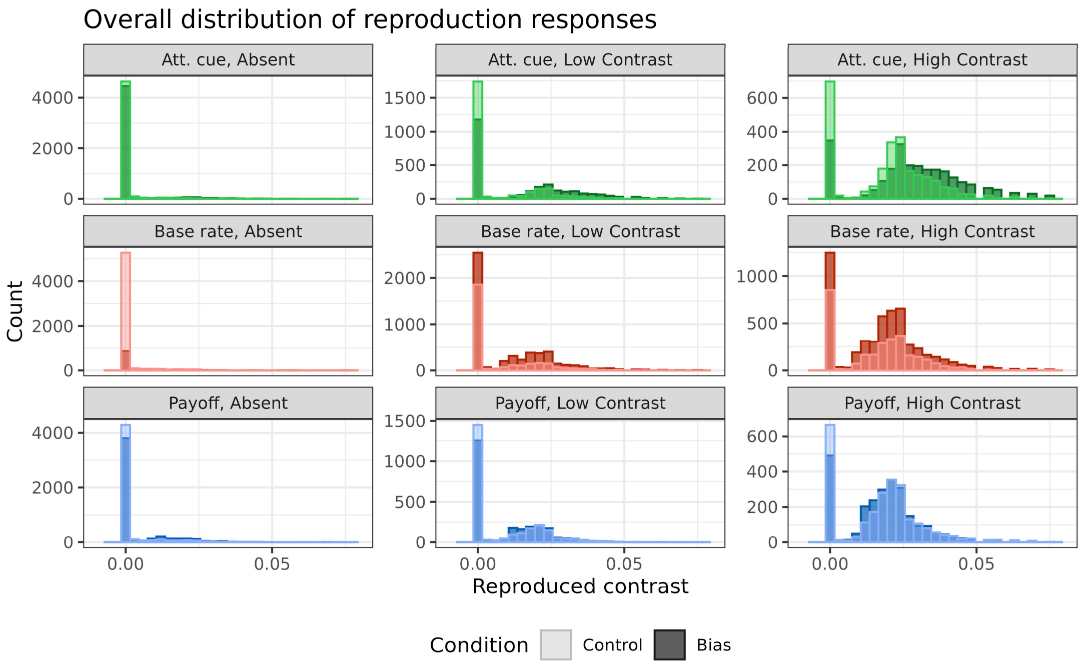

Figure S4: ***Experiment 2 distribution of all reproduction responses****. All the reproduction responses of all participants are plotted as a histogram.*

Supplementary Note S1: Hurdle-Gaussian modelling

Four Hurdle-gaussian models of increasing complexity were fit to the reproduction responses data using the *brms* R package (version 2.20.4) for Bayesian Multilevel Models[1]. For each model we ran four Markov chain Monte Carlo (MCMC) with 10,000 iterations each, 5,000 warm up iterations and a thinning factor of 1. All R-hat values were lower or equal to 1.02. The Hurdle-Gaussian model consists of a binary part (Bernoulli distribution) that indicates the probability of a given contrast value to pass the hurdle and a (Gaussian distribution) part that describes the values that went over the hurdle. For an outcome variable y (in our case a reproduction response), the model assumes that y will be zero or normally distributed depending on the probability of hu:

$$y \sim \begin{cases} 0 & \text{with probability hu,} \\ \text{Normal}(\mu, \sigma) & \text{with probability} 1 - \text{hu.} \end{cases}$$

Where μ is the mean of the normal distribution (mean *non-zero* reproduced contrast) and σ is the standard deviation of the normal distribution. hu is the hurdle component that indicates the probability of y being zero, modelled using Bernoulli distribution with logit link function. The likelihood function is:

$$\log P(y \mid \mu, \sigma, \text{hu}) = \begin{cases} \text{logit}(\text{hu}) & \text{if } y = 0, \\ \log(1 - \text{hu}) + \text{Normal}(y \mid \mu, \sigma) & \text{if } y > 0. \end{cases}$$

In the full model, response and hurdle depended on the interaction between the contrast of the target patch ($stim$) and condition (control or biased) ($cond$). We also allowed a random slope per participant and condition.

$$\text{Response: } y \sim \text{stim} \times \text{cond} + (\text{cond} \mid \text{participant})$$

$$\text{Hurdle: hu} \sim \text{stim} \times \text{cond} + (\text{cond} \mid \text{participant})$$

The intercept for the response term was assigned a prior of Student-t$(3,0,2.5)$. The intercept for hu was modeled with a prior of Logistic$(0,1)$. Flat priors were used for the fixed effects of stim, cond and their interaction stim × cond in both the response and hu components. For the random effects, the standard deviations of the intercepts and slopes for cond and their interactions with stim in both the response and hu components were specified with a prior of Student-t$(3,0,2.5)$, constrained to be non-negative. Additionally, the correlation structure among the random effects in the response and hu components was modeled using LKJ(1) distribution priors. The residual variance $sigma$ was assigned a prior of Student-t$(3,0,2.5)$, constrained to be non-negative.

Then, in the contrast model we fixed the hurdle parameter across conditions, meaning that it depended only on the contrast of the target patch ($stim$), whereas response depended on the interaction between the contrast of the target patch ($stim$) and condition ($cond$).

$$\text{Response: } y \sim \text{stim} \times \text{cond} + (\text{cond} \mid \text{participant})$$

$$\text{Hurdle: hu} \sim \text{stim} + (1 \mid \text{participant})$$

The intercept for the response term was assigned a prior of Student-t$(3,0,2.5)$. The intercept for hu was modeled with a prior of Logistic$(0,1)$. Flat priors were used for the fixed effects of stim, cond and their interaction stim × cond in the response component, and for stim in the hu component. For the random effects, the standard deviations of the intercepts and slopes for cond and their interactions with stim in the response component, and for stim in the in the hu component, were specified with a prior of Student-t$(3,0,2.5)$, constrained to be non-negative. Additionally, the correlation structure among the random effects in the response and hu components was modeled using LKJ(1) distribution priors. The residual variance $sigma$ was assigned a prior of Student-t$(3,0,2.5)$, constrained to be non-negative.

Inversely, in the hurdle model, response was fix across conditions and depended only on the contrast of the target patch ($stim$), whereas hurdle depended on the interaction between the contrast of the target patch ($stim$) and condition ($cond$).

$$\text{Response: } y \sim \text{stim} + (1 \mid \text{participant})$$

$$\text{Hurdle: hu} \sim \text{stim} \times \text{cond} + (\text{cond} \mid \text{participant})$$

The intercept for the response term was assigned a prior of Student-t$(3,0,2.5)$. The intercept for hu was modeled with a prior of Logistic$(0,1)$. Flat priors were used for the fixed effects of stim, cond and their interaction stim × cond in the hurdle component, and for stim in the response component. For the random effects, the standard deviations of the intercepts and slopes for cond and their interactions with stim in the hurdle component, and for stim in the in the response component, were specified with a prior of Student-t$(3,0,2.5)$, constrained to be non-negative. Additionally, the correlation structure among the random effects in the response and hu components was modeled using LKJ(1) distribution priors. The residual variance $sigma$ was assigned a prior of Student-t$(3,0,2.5)$, constrained to be non-negative.

Finally, in the baseline model, both response and hurdle depended only on the contrast of the target patch ($stim$).

$$\text{Response: } y \sim \text{stim} + (1 \mid \text{participant})$$

$$\text{Hurdle: hu} \sim \text{stim} + (1 \mid \text{participant})$$

The intercept for the response term was assigned a prior of Student-t$(3,0,2.5)$. The intercept for hu was modeled with a prior of Logistic$(0,1)$. Flat priors were used for the fixed effects of stim in both the response and hu components. For the random effects, the standard deviations of the intercepts and slopes for stim in both the response and hu components were specified with a prior of Student-t$(3,0,2.5)$, constrained to be non-negative. Additionally, the correlation structure among the random effects in the response and hu components was modeled using LKJ(1) distribution priors. The residual variance σ was assigned a prior of Student-t$(3,0,2.5)$, constrained to be non-negative.

Supplementary Note S2: Detection Hurdle regression

To test whether the rate of *zero* reproductions depended on observers' decision criterion in the detection task, we fit a linear mixed model using the logit-transformed proportion of present responses of the detection task and the logit-transformed probability of passing the hurdle in the reproduction task predicted by the full Hurdle-Gaussian model. The model was fit using the *brms* R package (version 2.20.4) for Bayesian Multilevel Models[1] and formally defined as follows:

$$\text{hu} \sim \text{answer} + (1 \mid \text{participant})$$

For a given contrast value, hu is the Hurdle-Gaussian predicted probability of passing the reproduction hurdle in the reproduction task, that is, the rate of *zero* to *non-zero* reproductions, whereas $answer$ is the probability of reporting said contrast value as 'present' in the detection task. Additionally, the model includes a random slope per participant to account for individual variability. The model had weakly informative priors. The prior for the fixed effect of answer was flat, whereas the intercept was assigned a prior of Student-t$(3,1.3,2.5)$. For the random effects, the standard deviations of the intercepts and slopes for participant, as well as the interaction between the intercept and participant, were specified with a prior of Student-t$(3,0,2.5)$, constrained to be non-negative. The residual variance σ was assigned a prior of Student-t$(3,0,2.5)$, constrained to be non-negative.

Supplementary Note S3: Experiment 2 results

When compared against the control condition, all manipulations (cue, base rate and payoff) increased the number of "present" responses, both in absent and present trials (see Sup. Figure S3). There was strong evidence (BF_{10} = 19, d = 0.44) for an effect in absent trials of the attentional cue condition and decisive evidence for an effect in all other conditions and contrast levels (BF_{10} > 100, d > 0.6; see Sup. Figure S3A). In the reproduction task, when compared against the control condition, the attentional cue resulted in medium to large positive effects (d > 0.5), meaning that observers reproduced patches with higher contrast when the cue was presented (BF_{10} > 80). In the base rate condition, the reproduced contrast was higher across all contrast levels when the stimulus-present trials were more likely ($d \geq 0.29$, $BF_{10} \geq 3$). Finally, in the payoff condition, reproductions were higher when misses were punished more than false alarms in absent (d = 0.55, BF_{10} = 178) and in low contrast trials (d = .34, BF_{10} = 4), but there was moderate evidence for no effect in the high contrast trials (d = .12, BF_{10} = 0.33; see Sup. Figure S3B for the exact Cohen's d and Bayes Factor values of each comparison). Overall, this pattern of results is qualitatively identical to the results of Experiment 1.

Next, we calculated the proportion of *zero* reproductions and the average *non-zero* reproduced contrast to independently evaluate the effects of each manipulation on reproduction hurdle and reproduced contrast. Similar to Experiment 1, the proportion of *zero* reproductions decreased as the contrast of the stimuli decreased. Likewise, the proportion of *zero* reproductions decreased when trials were cued (attentional cue manipulation), when the ratio of present to absent trials increased (base rate) or when misses were punished more strongly than false alarms (payoff; see Figure 5A). A Bayesian t-test revealed very strong evidence (d > 0.4, BF_{10} > 30) of an effect in all conditions across all contrast levels, except for the absent trials of the attentional cue condition and high contrast trials of the payoff condition, where the evidence for an effect was moderate (BF_{10} > 3). These results effectively confirm again that all manipulations increased the probability of reporting a *non-zero* contrast patch when compared to the control condition. Likewise, the *non-zero* reproductions were also similar to the results of Experiment 1 (see Figure 5B, see Sup. Figure S4 for the distributions prior to removing the zero-reproductions). The attentional cue resulted in medium to large effects across all contrast levels (d > 0.4, BF_{10} > 10). In contrast, in the base rate condition, there was very strong evidence (BF_{10} = 1.1e+04, d = 0.76) of an effect in absent trials, moderate evidence for no effect in low contrast trials (BF_{10} = 0.18, d = 0.04) and strong evidence for no effect in high contrast trials (BF_{10} = 0.08, d = -0.14) contrast trials. Lastly, in the payoff condition, there was moderate evidence for an effect in absent trials (BF_{10} = 5, d = 0.36) and moderate to strong evidence for no effect in low (BF_{10} = 0.11, d = -0.06) and high contrast trials (BF_{10} = 0.06, d = -0.23).

As before, to estimate whether the effect of the manipulations was driven by an effect in reproduced contrast or by a shift of the reproduction hurdle, we fitted four Hurdle-Gaussian models in which we either allowed both the contrast and hurdle parameters to vary across conditions (full model), only allowed the reproduced contrast parameter to

vary (contrast only model), only allowed the hurdle parameter to vary (hurdle only model), or we fixed both parameters across conditions (baseline model; see Sup. Note S1 for a detailed description of the modelling procedure). Using each model we then sampled new reproduction responses to assess whether only contrast, only hurdle or both parameters where necessary to recover the original effect on overall reproduced contrast (see Figure 5C). Similar to Experiment 1, in the attentional cue condition only the full model correctly predicts the empirical data across all contrast levels, whereas in the base rate and payoff conditions both the full and hurdle models are able to recreate the effect of the manipulations.

Next, following the same procedure as in Experiment 1, we compared all models against the baseline model and computed Bayes Factors for each comparison by assessing how well each model predicted the observed reproduction data (see Figure 5D). As in Experiment 1, the full model was the best performing model, suggesting that all manipulations influenced both the reproduction hurdle and the reproduced contrast ((log) $BF_{full-over-baseline} \geq 300$; (log) $BF_{full-over-second-best} \geq 40$; note that BF values are reported in log scale due to their magnitude). When directly comparing the contrast and hurdle models (see Figure 5E), there was decisive evidence ((log) $BF_{contrast-over-hurdle} = 47$) in favour of the contrast model in the attentional cue condition, whereas the opposite was true in the base rate ((log) $BF_{hurdle-over-contrast} = 175$) and payoff ((log) $BF_{hurdle-over-contrast} = 211$) conditions, suggesting that the attentional cue manipulation predominantly affects reproduced contrast, while the payoff and base rate conditions predominantly affect the reproduction hurdle.

Finally, as in Experiment 1, we tested whether the proportion of *zero* reproductions was dependent on the decision criterion of the detection task (see Figure 5F) by fitting linear mixed model using the effect of each manipulation over the proportion of absent responses in the detection task to predict the probability of *zero* reproductions (not passing the hurdle) in the reproduction task. Again, we found decisive evidence ($BF_{10} > 1000$, $r2 \geq 0.7$) of a positive association between the effect on the detection task and the predicted hurdle effect (see Figure 5G; see Sup. Note S2 for a detailed account of the analysis).

Supplementary Note S4: Participant removal criteria

Participants were outliers if their staircase thresholds (Experiment 1: two participants in the base rate condition; Experiment 2: one participant in the attentional cue condition and one participant in the base rate condition) signal detection theory d' or reproduction error (Experiment 1: two participants in the cue condition) fell outside four standard deviations from the sample mean across all conditions (cue, base rate and payoff) but independently for each experiment. Participants with a signal detection theory d' below zero were also removed (Experiment 1: five participants in the base rate condition and four participants in the payoff condition; Experiment 2: five participants in the base rate condition and three participants in the payoff condition). In total 13 participants were removed in Experiment 1, 10 participants in Experiment 2 and 1 participant in Experiment 3.

Supplementary Note S5: Experiment 1 and 2 instructions and practice procedure

Participants received instructions and performed extensive practice before starting the experiment. In Experiment 1, to ease participants into the detection task, they received instructions and then completed three practice blocks. First, 10 easy trials with trial-feedback and without performance requirements. Then two harder 20-trials blocks with an overall accuracy of at least 75%, first with trial-feedback and then only with block level feedback. Participants then went over the staircase block (see Methods - Staircase procedure). Participants then received instructions for the reproduction task and completed two practice blocks, first with trial-feedback and then only with block level feedback. Each practice block consisted of 20 trials, and they were required to get at least 15 correct trials. Finally, participants completed a 10-trials block where the detection and the reproduction task were interleaved. There were no performance requirements in the detection-reproduction practice block. In Experiment 2 the instructions and practice were largely the same except for the following details. The detection practice consisted of 3 blocks, 10 easy trials with trial-feedback and no minimum performance, 20 harder trials with trial-feedback and a required accuracy of 75%, and another 20-trials block with only block level feedback and a minimum accuracy of 75%. The reproduction practice consisted of three 20-trials blocks, first with trial-feedback and no performance requirement, then with trial-feedback and minimum accuracy of 75%, and then with block-level feedback and minimum accuracy of 75%. Reproduction responses were correct if they were within a range calculated for each contrast level. The lower boundary of the range was ((contrast ^ 0.7) / 2) ^ (1 / 0.7), and the upper boundary was ((contrast ^ 0.7) + (contrast ^ 0.7) / 2)) ^ (1 / 0.7). Finally, the detection-reproduction practice block consisted of 20 trials. After the tasks' instructions and practice, and just before the experimental trials started, participants in the payoff and base rate condition were instructed about the asymmetrical punishment (payoff condition) or stimulus prevalence scheme (base rate condition). Both in the payoff and base rate condition, participants completed an extra practice block that consisted of 25 trials with trial-by-trial feedback where either the payoff or base rate manipulation was in place to confirm they understood the instructions. In the attentional cue condition participants were explicitly told that the cue did not predict the presence of the target and that they should ignore it. Then they completed 20 practice cued trials. The experiment was divided into two blocks, one with the bias manipulation (attentional cue, base rate or payoff), and a control block. The order of the blocks was counterbalanced and roughly half of the participants started with the bias block. Instructions regarding the manipulations were delivered just before the bias block. For example, if a participant started with the control block of the payoff condition, they received the instructions regarding the differential punishment after having completed the control block, but if they started with the bias block, they received the manipulation instructions just before the first block.

Supplementary Note S6: Block level feedback rationale

Detection task feedback. To boost the efficacy of the bias manipulations in the detection task, participants received response-specific block-level feedback in the payoff and base rate condition, meaning that they were informed about the exact number of incorrect "absent" and "present" responses, thus allowing participants to be aware of the stimulus-response contingency of the manipulations. Participants did not receive such response-specific feedback in the attentional cue condition, as this was unnecessary for the manipulation to be effective. Furthermore, such feedback might have had adverse effects on the efficacy of the manipulation by allowing them to correct for any biased performance due to the perceptual effect of the cue itself. Instead, the attentional cue condition only contained general feedback about the total number of incorrect detection responses (an example of the block-level feedback is provided in Sup. Note S7 and S8).

Reproduction task feedback. To prevent a direct effect of feedback on the reproduction responses, feedback about performance in the reproduction task was always the same for all bias conditions. In Experiment 1, participants were only informed about their overall performance in the reproduction task, without specifying the direction of the error (contrast under- or over-estimation). Instead, the feedback consisted of the overall number of reproductions that were too off-track, merely to keep participants motivated in performing the task as well as they could. To evaluate the correctness of reproduction responses we used a threshold based on pilot data that yield a 75% correct responses rate. In absent trials reproduction responses were correct if they were equal to zero, whereas in present trials the reproduction patch contrast had to be higher than a threshold that depended on the target patch contrast in present trials. The threshold was calculated by transforming the target patch contrast into a linear scale by raising it to the power of 0.7[82], then dividing it by 2 and then bringing back the value to raw contrast scale by raising it to the power of $\frac{1}{0.7}$ (see formula below where *tc* stands for target patch contrast).

$$\left(\frac{\mathrm{tc}^{0.7}}{2}\right)^{\frac{1}{0.7}}$$

In Experiment 2 and 3, to further eliminate any potential effect of feedback on reproduction responses, participants did not receive feedback on their reproduction responses in any of the conditions.

Supplementary Note S7: Experiment 1 block level performance feedback

In all conditions participants were informed about their progress and the number of incorrect responses in each task. In the payoff (*top panel*) and base rate (*middle panel*) conditions participants were informed about the specific number of misses and false alarms. Both payoff and base rate block level feedback were almost identical, except for the cost for each mistake in the detection task. In the attentional cue condition (*bottom panel*) participants were informed about the overall number of incorrect responses in both the detection and in the reproduction task.

You have completed 10% of the experiment.
You are doing well!
This was your performance:

You missed 5 patches (-5 each), so you lost 25 points.
You made 5 false alarm(s) (-1 each), so you lost 5 points.
Your reproductions were too off track 5 times, you lost 5 points.

Press any response button to continue.

You have completed 10% of the experiment.
You are doing well!
This was your performance:

You missed 5 patches (-1 each), so you lost 5 points.
You made 5 false alarm(s) (-1 each), so you lost 5 points.
Your reproductions were too off track 5 times, you lost 5 points.

Press any response button to continue.

You have completed 10% of the experiment.
You are doing well!
This was your performance:

You gave 10 incorrect answers and you lost 10 points.
Your reproductions were too off track 5 times, you lost 5 points.

Press any response button to continue.

Supplementary Note S8: Experiment 2 block level performance feedback

In all conditions participants were informed about their progress and the number of incorrect responses in the detection task. In the payoff (*top panel*) and base rate (*middle panel*) conditions participants were informed about the specific number of incorrect present and absent responses. Both payoff and base rate block level feedback were almost identical, except for the cost for each type of mistake in the detection task. In the attentional cue condition (*bottom panel*) participants were informed about the overall number of incorrect responses in both the detection and in the reproduction task.

You have completed 10% of the experiment.
This was your performance during the last 50 trials.

You incorrectly answered present 5 times (-5 each), you lost 25 points.
You incorrectly answered absent 5 times (-1 each), you lost 5 points.

Press any response button to continue.

You have completed 10% of the experiment.
This was your performance during the last 50 trials.

You incorrectly answered present 5 times (-1 each), you lost 5 points.
You incorrectly answered absent 5 times (-1 each), you lost 5 points.

Press any response button to continue.

You have completed 10% of the experiment.
This was your performance during the last 50 trials.

You made 10 mistakes (-1 each), you lost 10 points.

Press any response button to continue.

References

1. Bürkner, P.-C. Brms: An R Package for Bayesian Multilevel Models Using Stan. *Journal of Statistical Software* **80**, 1–28 (2017).
