# Supplementary material for: The act of detecting a stimulus contaminates measures of conscious experience with decision biases
Source: Nat Commun. 2026 May 8;17:6254. doi: 10.1038/s41467-026-72567-6 (PMC13376516; doi:10.1038/s41467-026-72567-6)
Supplement: Supplementary file 2 — Reporting Summary [file 41467_2026_72567_MOESM2_ESM.pdf]

nature portfolio

Corresponding author(s): Nicolás Sánchez-Fuenzalida

Last updated by author(s): Mar 26, 2026

Reporting Summary

Nature Portfolio wishes to improve the reproducibility of the work that we publish. This form provides structure for consistency and transparency in reporting. For further information on Nature Portfolio policies, see our Editorial Policies and the Editorial Policy Checklist.

Statistics

For all statistical analyses, confirm that the following items are present in the figure legend, table legend, main text, or Methods section.

n/a	Confirmed	
☐	☒	The exact sample size (*n*) for each experimental group/condition, given as a discrete number and unit of measurement
☐	☒	A statement on whether measurements were taken from distinct samples or whether the same sample was measured repeatedly
☐	☒	The statistical test(s) used AND whether they are one- or two-sided<br>*Only common tests should be described solely by name; describe more complex techniques in the Methods section.*
☒	☐	A description of all covariates tested
☒	☐	A description of any assumptions or corrections, such as tests of normality and adjustment for multiple comparisons
☐	☒	A full description of the statistical parameters including central tendency (e.g. means) or other basic estimates (e.g. regression coefficient) AND variation (e.g. standard deviation) or associated estimates of uncertainty (e.g. confidence intervals)
☒	☐	For null hypothesis testing, the test statistic (e.g. *F*, *t*, *r*) with confidence intervals, effect sizes, degrees of freedom and *P* value noted<br>*Give P values as exact values whenever suitable.*
☐	☒	For Bayesian analysis, information on the choice of priors and Markov chain Monte Carlo settings
☐	☒	For hierarchical and complex designs, identification of the appropriate level for tests and full reporting of outcomes
☐	☒	Estimates of effect sizes (e.g. Cohen's *d*, Pearson's *r*), indicating how they were calculated

Our web collection on statistics for biologists contains articles on many of the points above.

Software and code

Policy information about availability of computer code

Data collection	Data was collected using a script made using Python 3.8.
Data analysis	Data analysis was executed using scripts written in R. All data and analysis scripts are available in the repository linked in the next section.

For manuscripts utilizing custom algorithms or software that are central to the research but not yet described in published literature, software must be made available to editors and reviewers. We strongly encourage code deposition in a community repository (e.g. GitHub). See the Nature Portfolio guidelines for submitting code & software for further information.

Data

Policy information about availability of data

All manuscripts must include a data availability statement. This statement should provide the following information, where applicable:

- Accession codes, unique identifiers, or web links for publicly available datasets
- A description of any restrictions on data availability
- For clinical datasets or third party data, please ensure that the statement adheres to our policy

All data (raw and processed) is available in https://osf.io/tbfne/

Research involving human participants, their data, or biological material

Policy information about studies with human participants or human data. See also policy information about sex, gender (identity/presentation), and sexual orientation and race, ethnicity and racism.

Reporting on sex and gender	Both sex and gender were not relevant for the current study. Self-reported gender was collected for each experiment merely for descriptive reasons. Total counts per experiment are reported.
Reporting on race, ethnicity, or other socially relevant groupings	There are no race, ethnicity, or other socially relevant groupings that are relevant for this study.
Population characteristics	Nothing to add here.
Recruitment	Participants were recruited through an ad published in the University of Amsterdam Laboratory website. The ad was available to anyone registered in the Laboratory website. The pool is composed mostly of undergraduate students. Recruiting undergraduate students is a common practice in experiment psychology research.
Ethics oversight	All three experiments were approved by University of Amsterdam Ethics Review Board and the Ethics committee of the Vrije Universiteit Amsterdam.

Note that full information on the approval of the study protocol must also be provided in the manuscript.

Field-specific reporting

Please select the one below that is the best fit for your research. If you are not sure, read the appropriate sections before making your selection.

☐ Life sciences ☒ Behavioural & social sciences ☐ Ecological, evolutionary & environmental sciences

For a reference copy of the document with all sections, see nature.com/documents/nr-reporting-summary-flat.pdf

Behavioural & social sciences study design

All studies must disclose on these points even when the disclosure is negative.

Study description	This study consisted of three quantitative experiments with 3 between-subjects bias manipulations (attentional cue, base rate and payoff) and 2 within-subject bias conditions (no-bias and biased).
Research sample	251 participants took part in experiment 1, 168 in experiment 2, and 109 in experiment 3. Out of these participants, 238 (187 women, 46 male and 5 other; average age 20.8, SD = 2.98), 158 (131 women, 24 male and 3 other; average age 21, SD = 2.9) and 109 (94 women, 13 men and 2 other; average age 19.9, SD = 2.25) participants were analyzed, respectively.
Sampling strategy	The sample size of experiment 1 was determined by applying an optional stopping rule that depended on the main comparisons of interest, whereas for experiment 2 we conducted a sequential sampling plan based on the effect sizes of experiment 1. In experiment 1 we collected the data of 30 participants, removed outliers and ran a Bayesian t-test between the control and biased condition in the decision (rate of 'present' responses) and reproduction task (reproduced contrast). These tests follow the same procedure as the main hypothesis tests as reported in Figure 1C and D. For each comparison, if there was at least moderate evidence for the presence or absence of an effect (for either the null or the alternative hypothesis; BF10 > 3 or BF10 < 0.33) in both tasks we stopped data collection, otherwise we collected five more subjects and repeated the process. In this framework optional stopping or data peaking is not considered problematic (Rouder, 2014). In experiment 2, we aimed to collect a sample that allowed us to detect at least the smallest effect size found in experiment 1 (Cohen's d = 0.4) with at least moderate evidence (BF10 >= 3 or BF10 ≤ 0.33). We used the BFDA R package (Schönbrodt & Stefan, 2019) for Bayesian Design Analysis (Schönbrodt & Wagenmakers, 2018). A sequential sampling plan indicated that a sample of 50, 65 and 85 participants would yield respectively a power of 70%, 80% and 90% to detect the aforementioned effect. We decided to collect the data of at least 50 participants and continue testing until all comparisons yielded moderate evidence for the null or alternative hypothesis (BF10 >= 3 or BF10 ≤ 0.33), or until we collected 85 participants (50 and 85 refer to the participant count after filtering outliers). In experiment 3 we aimed to collect the same number of participants as in experiment 2 after removing outliers.
Data collection	Data was collected using a computer and a script made using Python and Psychopy. Participants were received by a researcher and then taken to a closed cubicle were they completed the experiment by themselves. Researchers were not blind to the hypothesis nor the study hypothesis during data collection.
Timing	The data of Experiment 1 was collected between August and November of 2021. The data of Experiment 2 was collected between September and October 2022 and between November 2023 and June 2024. The data of Experiment 3 was collected between November 2024 and February 2025.
Data exclusions	Participants were outliers and removed if their staircase thresholds (experiment 1: two participants in the base rate condition; experiment 2: one participant in the attentional cue condition and one participant in the base rate condition) signal detection theory d' or reproduction error (experiment 1: two participants in the cue condition) fell outside four standard deviations from the sample mean across all conditions (cue, base rate and payoff) but independently for each experiment. Participants with a signal detection

	theory d' below zero were also removed (experiment 1: five participants in the base rate condition and four participants in the payoff condition; experiment 2: five participants in the base rate condition and three participants in the payoff condition). In total 13 participants were removed in experiment 1, 10 participants in experiment 2 and 1 participant in experiment 3.
Non-participation	In Experiment 1, 10 participants did not understand the instructions and therefore did not continue to the experiment. Similarly, in Experiment 2, 13 participants did not understand the instructions and did not continue to the experiment. In Experiment 3, all participants finished the experiment.
Randomization	Participants were allocated to one of the bias manipulations conditions (attentional cue, base rate or payoff) as they were recruited. For example, participant 1 was assigned to the attentional cue condition, participant 2 to the base rate condition and so on and so forth. Within each bias condition (attentional cue, base rate or payoff) participants were assigned to start either the control or the biased condition.

Reporting for specific materials, systems and methods

We require information from authors about some types of materials, experimental systems and methods used in many studies. Here, indicate whether each material, system or method listed is relevant to your study. If you are not sure if a list item applies to your research, read the appropriate section before selecting a response.

Materials & experimental systems

n/a	Involved in the study
☒	☐ Antibodies
☒	☐ Eukaryotic cell lines
☒	☐ Palaeontology and archaeology
☒	☐ Animals and other organisms
☒	☐ Clinical data
☒	☐ Dual use research of concern
☒	☐ Plants

Methods

n/a	Involved in the study
☒	☐ ChIP-seq
☒	☐ Flow cytometry
☒	☐ MRI-based neuroimaging

Plants

Seed stocks	NA
Novel plant genotypes	NA
Authentication	NA
