# Supplementary material for: The act of detecting a stimulus contaminates measures of conscious experience with decision biases
Source: Nat Commun. 2026 May 8;17:6254. doi: 10.1038/s41467-026-72567-6 (PMC13376516; doi:10.1038/s41467-026-72567-6)
Supplement: Supplementary file 3 — Transparent Peer Review file [file 41467_2026_72567_MOESM3_ESM.pdf]

nature portfolio

Peer Review File

The act of detecting a stimulus contaminates measures of conscious experience with decision biases

Corresponding Author: Dr Nicolás Sánchez-Fuenzalida

This file contains all reviewer reports in order by version, followed by all author rebuttals in order by version.

Version 0:

Reviewer comments:

Reviewer #1

(Remarks to the Author)
The manuscript investigates conscious perception in a large sample of participants by contrasting results from detection and reproduction tasks under several contextual manipulations, namely attention cues, asymmetrical base rates, and payoff schemes. The main focus is on assessing whether reproduction responses can provide a less biased assessment of conscious perceptual reports than detection tasks, and whether detection tasks introduce systematic biases that depend on contextual and task design factors. The modeling approach allows the authors to disentangle post-perceptual shifts in reproduction behavior from effects on conscious experience, while also demonstrating systematic biases associated with detection tasks.

Overall, the manuscript is clearly written, the research question is highly relevant to the study of conscious perception, and the findings appear robust, convincing, and broadly important. I do not have methodological concerns about the current approach, but I have several major points regarding the conceptual take-home message and a few other details.

First, the main conclusion appears to be that reproduction tasks, rather than detection tasks, may be better suited to assess conscious experience. However, it is unclear exactly how this would be implemented in practice. In reproduction tasks, the stimulus is usually always presented, and the participant responds along a continuous scale, whereas Yes/No detection tasks involve explicit presence/absence judgments. How would the authors recommend assessing conscious perception in purely reproduction or adjustment tasks? Specifically, what would be the proposed method for identifying trials with or without conscious perception of the reproduced feature? I think, making this clearer would strengthen the contribution of the paper.

Second, it is unclear whether reproduction responses (even in the control condition) are also biased in trials where participants report “present,” potentially leading to systematic overestimations of the reproduced feature.

Third, I found the term “detection threshold shift” a bit misleading when referring to a post-perceptual bias rather than a genuine change in sensitivity.

In the introduction, reproduction tasks are presented as the better method, but the rationale for this claim is not well developed. Reproduction tasks are themselves susceptible to multiple types of biases, so it would be important to explain why, in this specific experimental context, they might nonetheless be preferred—ideally already in the opening of the paper.
Finally, I wonder whether a factorial analytical approach (e.g., ANOVA with main effects and interactions) might have been more appropriate than multiple pairwise t-tests.

Minor:
Figure 2, panel C: “contrast” is misspelled as “conctrast.”

(Remarks on code availability)

Reviewer #2

(Remarks to the Author)
In this manuscript, the authors aim to disentangle perceptual from decisional factors by comparing how contextual factors (attentional cues, base rates, and payoffs) impact performance and bias using two tasks: a detection task (target present/absent) and a reproduction task (reporting perceived contrast.) Behavioral results and Hurdle–Gaussian modeling suggest that attentional cues modulated perception, and base-rate and payoff manipulations primarily induced decision biases that also affect reproduction results. The authors suggest that stimulus detection influences reports in reproduction tasks, and that reproduction tasks should be preferred to detection tasks when measuring conscious perception. The manuscript is well written, and the experiments, modelling and logic of the approach are generally clear. However, there are several issues that should be addressed to improve the manuscript.

The manuscript falls between a rather narrow focus, centered on a specific combination of a detection and reproduction task, and much broader theoretical claims regarding the distinction between perceptual and decisional processes. It would be good to better define the intended scope of the current findings. While the title suggests a relatively specific topic, the broader goal seems to imply implications for the study of perception and bias in general.

Whether the approach successfully dissociates perception and decision bias is not clear. While there is strong evidence for changes in criterion with attentional cues, base rate manipulations, and different payoffs, and many studies have shown that attention may change appearance, whether perceived contrast actually changed in the attention condition remains unclear. In this context, it would be good to discuss studies that suggest that perceived contrast is not changed by attention (for example, Schneider, 2006, 2011, 2021; Schneider & Komlos, 2008). Regarding the difference between the attentional cue and the other two conditions in Experiments 1 and 2: do we know whether the change in decision criterion influenced reports in the reproduction task or is the interpretation of these results only meaningful when also considering the results of the control experiment? Related to that, to what extent were participants aware of their false alarms/ liberal criteria, and what is the influence of that on zero/non-zero responses? Confidence ratings and reaction times could be helpful in this context as well. These points could be discussed in more detail in the General Discussion, possibly discussing a broader perspective on methodological alternatives (e.g., two-alternative forced choice tasks, confidence reports etc.), and implications for consciousness research.

A key contribution of this study seems to be to disentangle perceptual effects from decisional biases. However, that is not clear throughout the paper, including the abstract concluding with “We propose that reproduction is better suited to assay conscious experience than detection” and the general discussion. It could be stated more explicitly how this approach can disentangle the two beyond the paradigm used here. Specifically, how applicable are these findings for studies of conscious perception more broadly and how successful is the model in determining whether a stimulus was consciously experienced, especially in more ambiguous cases?

It seems that Experiment 1 had initially a different goal, as the authors write "However, we realized that an increase in the average reproduced contrast as reported in Figure 1D may in fact result from two distinct influences on reproduction behaviour: on the one hand, the overall average reproduced contrast reflects the reproduced contrast on non-zero contrast reproductions, but on the other hand, it also reflects the ratio of zero to non-zero contrast reproductions." Given that several issues in Experiment 1 were addressed in Experiment 2, it might have been clearer to present Experiment 2 as the main experiment—based on the exploratory analysis of Experiment 1, especially considering the rather detailed description of Experiment 1.

While most parts are explained well in high detail, certain parts remain unclear.
For example, in the results section, the authors write: "Each of these conditions which was accompanied by a neutral control condition (see Figure 1B for a graphical summary of the manipulations)." However, Figure 1B does not clearly explain what the neutral condition exactly is.

It could be said more explicitly how the control experiment shows the following: "Crucially, this control experiment shows that the effect the base rate had on the proportion of zero reproductions in experiments 1 and 2 disappeared when removing the detection context during reproduction, clearly favouring the interpretation that attentional cues influence subjectively perceived contrast (conscious perception), while the effect of base rate is non-perceptual in nature."

What type of "different" pattern was expected? "If the detection task had not influenced how participants behaved in the reproduction task, the pattern of results should not be affected when removing it. However, if the detection task inadvertently affected how participants respond in the reproduction task, the reproduction task without detection would be expected to show a different pattern of results despite identical physical stimulation."

Explicitly mention why there was a big difference in the number of participants: "In experiment 3 we aimed to collect the same number of participants as in experiment 2 after removing Outliers."

When d′ is mentioned, the curly quotation mark appears to be used instead of the straight apostrophe.

The legends of the figures could be improved.

Introduce and define clearly terms that might not be familiar to the average readers of the journal, for example, define zero

and non-zero reproductions; occasional dense passages may lose readers less familiar with the topic.

(Remarks on code availability)

Reviewer #3

(Remarks to the Author)

The study "The act of detecting a stimulus contaminates measures of conscious experience with unrelated cognitive factors" seeks to distinguish perceptual from decision effects in consciousness judgments. Participants viewed briefly presented with Gabor patches that varied in contrast (low, high) across trials. In experiments 1 and 2, participants were asked to perform either a detection task (present or absent) or a reproduction task (adjusting the contrast of the Gabor to match their perception). Importantly, the study introduced three types of bias (manipulated between participants), a base rate bias, a payoff bias, and an attentional cue bias. The results of the first two experiments showed that although all bias conditions elicited more "present" responses in the detection task, only the attentional cue condition resulted in an increase in perceived contrast in the reproduction task for detected targets. The authors conclude that the base rate and payoff conditions induced decision bias whereas the attentional cue resulted in an actual perceptual shift. Modeling the data using a Hurdle-Gaussian model supported the presence of both types of bias in the data. A control experiment suggested that detection tasks may induce decision bias, therefore contaminating measures of conscious experience.

The method of the study is novel and the results very interesting, making a contribution to the ongoing debate about how to best measure conscious experience. I have a few comments about the methodology and interpretation of the results.

1. To me the results show a clear perceptual effect in the attentional cue condition. However, it is still worth thinking whether the results in the base rate and payoff conditions are purely decision-based.

If bias is purely decision based, this should be reflected in the non-zero contrast reproduction performance across contrasts. Let's take the base rate condition for example. We expect that in the control condition, there would be a difference between reproduced contrast in the high compared to the low contrast condition because these judgments largely contain truly perceived trials. However, in the bias condition, the difference between low and high contrast conditions should be affected by not only truly perceived trials (which differed in perceived contrast), but also trials in which participants responded without having any true perceptual experience (which presumably elicited similar reproduced contrast across contrast conditions because nothing was perceived). In other words, in the control condition the comparison between high and low contrast reflects "signal" while in the biased condition the same comparison reflects "signal" plus some "noise" which should be of similar magnitude across contrast levels. I wonder if the authors could address whether the difference between high and low contrast conditions are consistent with the idea of "noise" (which would be consistent with decision bias) or not (which would be consistent with a perceptual account).

2. The proposed two-step model, binary (threshold) followed by a linear (contrast) judgments, makes predictions about reaction time. For example , as I understand it, RT for zero contrast judgments would be faster because on these trials processing end after the first step. There are of course more predictions one can make. It would be interesting to look at RT results and also investigate differences in Experiment 3 in which target processing presumably changes in the absence of the detection task.

3. The title could be improved. Unrelated cognitive factors sounds a bit too general. The title seems to disproportionately emphasize experiment 3 rather than represent the entire study.

4. The fact that the tasks were manipulated between subjects was not immediately clear to me. I eventually found it in the methods. It would be good to clarify earlier/more clearly in the manuscript and figure. The fact that the N is so uneven between conditions (and much higher for the condition that shows the perceptual effect in Experiment 1) also raises a flag and should be addressed.

5. Since the authors emphasize the results of experiment 3, this experiment should have its own clearly marked section. It would be helpful to include more discussion on how the absence of a detection task changes the processing of the stimulus (replacing the two step model) and how zero-contrast judgments may arise from qualitatively different processes compared to "absent" responses in the detection task.

6. Line 496: cued trials resulted in a higher proportion of zero reproductions across all contrast levels" should that be "lower", or am I misunderstanding?

(Remarks on code availability)
I did not try to run the code but I can access the Github with all the files.

Version 1:

Reviewer comments:

Reviewer #1

(Remarks to the Author)
The authors have addressed my main concerns, but one part added in response to my comments remains misleading. Specifically, the manuscript now suggests that attractive serial dependence, one of the classic sources of bias in reproduction tasks (and also in forced-choice tasks), is purely perceptual. The current state of the literature points in the opposite direction: attractive history biases are generally understood to arise largely, if not predominantly, at post-perceptual stages. More appropriate references for reviews and key findings include (Barbosa et al., 2020; Ceylan et al., 2021; Fischer et al., 2024; Fritsche et al., 2017; Luo et al., 2025; Pascucci et al., 2019, 2023; Sadil et al., 2023; Sheehan & Serences, 2022) (to mention but a few). Of course I am not requesting to cite all these references, just to give a better sense of the current state of knowledge and I therefore strongly encourage the authors to frame this section in accordance with what is actually known from the broader empirical literature rather than relying on a single opinion.
In addition, history-dependent biases are not the only sources of systematic errors in reproduction responses. Well-documented effects such as regression to the mean and stimulus-specific biases, whose expression depends on the structure of the stimulus space (e.g., linear, constrained, circular), also contribute, and these too are likely to involve post-perceptual processing (see also 'swap errors' in the domain of working memory research).
Related to this, the conclusion that reproduction tasks are sensitive to perceptual manipulations but not to post-perceptual ones is misleading. Several studies, for instance, show clear influences of post-perceptual manipulations such as memory load and feedback on reproduction performance and biases (Balikou et al., 2015; Markov et al., 2024).
For these reasons, if, in addition to the argument of avoiding a binary threshold, the authors' motivation for preferring reproduction responses over forced-choice judgments is based on a presumed selective targeting of perceptual rather than post-perceptual processes, I find this argument unconvincing and potentially misleading.

References
Balikou, P., Gourtzelidis, P., Mantas, A., Moutoussis, K., Evdokimidis, I., & Smyrnis, N. (2015). Independent sources of anisotropy in visual orientation representation: A visual and a cognitive oblique effect. Experimental brain research, 233(11), 3097–3108.
Barbosa, J., Stein, H., Martinez, R. L., Galan-Gadea, A., Li, S., Dalmau, J., Adam, K. C. S., Valls-Solé, J., Constantinidis, C., & Compte, A. (2020). Interplay between persistent activity and activity-silent dynamics in the prefrontal cortex underlies serial biases in working memory. Nature Neuroscience, 23(8), Articolo 8. https://doi.org/10.1038/s41593-020-0644-4
Ceylan, G., Herzog, M. H., & Pascucci, D. (2021). Serial dependence does not originate from low-level visual processing. Cognition, 212, 104709. https://doi.org/10.1016/j.cognition.2021.104709
Fischer, C., Kaiser, J., & Bledowski, C. (2024). A direct neural signature of serial dependence in working memory. eLife, 13. https://doi.org/10.7554/eLife.99478.1
Fritsche, M., Mostert, P., & de Lange, F. P. (2017). Opposite effects of recent history on perception and decision. Current Biology, 27(4), 590–595.
Luo, M., Zhang, H., Fang, F., & Luo, H. (2025). Reactivation of previous decisions repulsively biases sensory encoding but attractively biases decision-making. PLOS Biology, 23(4), e3003150. https://doi.org/10.1371/journal.pbio.3003150
Markov, Y. A., Tiurina, N. A., & Pascucci, D. (2024). Serial dependence: A matter of memory load. Heliyon, 10(13), e33977. https://doi.org/10.1016/j.heliyon.2024.e33977
Pascucci, D., Mancuso, G., Santandrea, E., Della Libera, C., Plomp, G., & Chelazzi, L. (2019). Laws of concatenated perception: Vision goes for novelty, decisions for perseverance. PLOS Biology, 17(3), e3000144. https://doi.org/10.1371/journal.pbio.3000144
Pascucci, D., Tanrikulu, Ö. D., Ozkirli, A., Houborg, C., Ceylan, G., Zerr, P., Rafiei, M., & Kristjánsson, Á. (2023). Serial dependence in visual perception: A review. Journal of Vision, 23(1), 9. https://doi.org/10.1167/jov.23.1.9
Sadil, P., Cowell, R. A., & Huber, D. E. (2023). The push–pull of serial dependence effects: Attraction to the prior response and repulsion from the prior stimulus. Psychonomic Bulletin & Review, 1–15.
Sheehan, T. C., & Serences, J. T. (2022). Attractive serial dependence overcomes repulsive neuronal adaptation. PLoS biology, 20(9), e3001711.

(Remarks on code availability)

Reviewer #2

(Remarks to the Author)
With the changes and additions, the manuscript has improved overall. While the revised manuscript discusses potential broader implications of the results, the scope remains somewhat vague. It would strengthen the manuscript to explicitly define the scope earlier in the text, clarifying to what extent the findings go beyond contrast detection or discrimination with simple Gabor stimuli and the parameters chosen here. This should include a discussion of the limitations due to the types of stimuli suitable for reproduction tasks, while also acknowledging that reproduction tasks, and more generally appearance-based tasks, are already common in the consciousness literature. Avoiding potential overgeneralization when interpreting the broader applicability of the results seems important.
More specifically, a major issue concerns the role of parameters in reproduction tasks that include no-stimulus trials. Over the course of a reproduction experiment, there is an inherent implicit detection component that varies with the proportion of

no-stimulus trials, the presented stimulus strengths, and, importantly, the frequency of non-zero responses. These factors can confound what is considered “conscious perception.” This issue should be discussed explicitly and in more depth, including reference to the limits of the reproduction approach itself.
Statements suggesting that traditional methods cannot disentangle perceptual and non-perceptual effects, but reproduction tasks with no-stimulus trials do, may overstate the certainty of the conclusions. Concerns about detection paradigms are well known. The statement “This highlights the need for caution in using and interpreting results relying on detection judgments, despite their central role in consciousness research” seems to overemphasize caution for detection paradigms, contrasting them with reproduction paradigms. Similar caution may be warranted when interpreting results of reprduction tasks, including zero responses in these tasks, as they can also reflect implicit decision criteria rather than the absence of perceptual experience per se.
The abstract and a few sections in the manuscript remain somewhat difficult to follow for a general readership. For example, the connection between the “mere presence of a detection context” and attentional cues, priors, or payoff structures is not immediately clear. Phrases such as “truly experience” are vague. Additionally, simplifying the language throughout and avoiding overcomplicated expressions would enhance readability.

(Remarks on code availability)

Reviewer #3

(Remarks to the Author)
The authors have sufficiently addressed my comments in this revision. I find the manuscript, suitable for publication.

(Remarks on code availability)

Version 2:

Reviewer comments:

Reviewer #1

(Remarks to the Author)
The authors have done a remarkable job revising the manuscript. I only have one remaining comment, aimed at avoiding unnecessary confusion. When discussing serial dependence, the authors now make it a relatively central point in the Introduction, but some statements remain unclear. For example, “The repulsive effect in orientation reproduction suggests that previous decisions can have non-perceptual effects on a subjective measure like reproduction,” appears to conflict with earlier arguments that interpret the repulsive effect as perceptual. Given that the nature of both attractive and repulsive serial dependence effects is still actively debated, I would avoid emphasizing this distinction in terms of processing stages too strongly.
My initial comment, which I still consider important, was that reproduction responses are not a direct measure of perception per se, as they are susceptible to multiple post-perceptual influences. These include post-perceptual components of serial dependence effects, but also memory-related biases, category boundary effects, regression to the mean, and other decision-level factors. I think the relevant section could be reduced to this more general point, while still generally referring to serial dependence as an example where post-perceptual factors plays a role.

(Remarks on code availability)

Reviewer #2

(Remarks to the Author)
The changes clearly improved the manuscript. Experiment 3 shows very few non-zero reproductions in the target-absent trials, suggesting that the bias is indeed absent or weak under these specific conditions. However, an implicit detection component is likely still present. It is unclear whether different experimental parameters would allow this component to influence reproduction responses, even in the absence of an explicit detection task, for example with different proportions of zero/non-zero responses, with other parameters varied, or other stimuli/ stimulus dimensions. While the authors now address these limits and refer to future work that would need to replicate these findings, it would be helpful to provide a clear rationale for why the same effect should be expected under other conditions, and not be limited to the specific design, stimuli, and procedure used here.

(Remarks on code availability)

Open Access This Peer Review File is licensed under a Creative Commons Attribution 4.0 International License, which permits use, sharing, adaptation, distribution and reproduction in any medium or format, as long as you give appropriate credit to the original author(s) and the source, provide a link to the Creative Commons license, and indicate if changes were made.
In cases where reviewers are anonymous, credit should be given to 'Anonymous Referee' and the source.
The images or other third party material in this Peer Review File are included in the article’s Creative Commons license, unless indicated otherwise in a credit line to the material. If material is not included in the article’s Creative Commons license and your intended use is not permitted by statutory regulation or exceeds the permitted use, you will need to obtain permission directly from the copyright holder.
To view a copy of this license, visit https://creativecommons.org/licenses/by/4.0/

Reviewer's comments

Reviewer #1 (Remarks to the Author):

The manuscript investigates conscious perception in a large sample of participants by contrasting results from detection and reproduction tasks under several contextual manipulations, namely attention cues, asymmetrical base rates, and payoff schemes. The main focus is on assessing whether reproduction responses can provide a less biased assessment of conscious perceptual reports than detection tasks, and whether detection tasks introduce systematic biases that depend on contextual and task design factors. The modelling approach allows the authors to disentangle post-perceptual shifts in reproduction behaviour from effects on conscious experience, while also demonstrating systematic biases associated with detection tasks.

Overall, the manuscript is clearly written, the research question is highly relevant to the study of conscious perception, and the findings appear robust, convincing, and broadly important. I do not have methodological concerns about the current approach, but I have several major points regarding the conceptual take-home message and a few other details.

1. First, the main conclusion appears to be that reproduction tasks, rather than detection tasks, may be better suited to assess conscious experience. However, it is unclear exactly how this would be implemented in practice. In reproduction tasks, the stimulus is usually always presented, and the participant responds along a continuous scale, whereas Yes/No detection tasks involve explicit presence/absence judgments. How would the authors recommend assessing conscious perception in purely reproduction or adjustment tasks? Specifically, what would be the proposed method for identifying trials with or without conscious perception of the reproduced feature? I think, making this clearer would strengthen the contribution of the paper.

Author responses in green

We want to first thank Reviewer #1 for taking the time to read our manuscript and for the helpful comments provided. We believe the revised document is a better manuscript because of them.

We have revised the manuscript to include a new section (lines 658-684 in tracked_changes.pdf) where we describe how the reproduction approach can be used in consciousness research. Although traditional reproduction tasks rely on the presentation of a stimulus in all trials, we show in our study that this setup can be extended to include both absent and present trials. In this context reproduction responses reflect the subjective strength of the perceptual experience: *non-zero* reproductions indicate some perceptual experience graded in a continuous fashion, whereas *zero* reproductions reflect its absence. We also highlight how this approach can be combined with manipulations of awareness (masking, attentional blink, continuous flash suppression, etc.) to study the difference in the processes that underlie consciously perceived stimuli and stimuli that remains unconscious; how reproduction responses can be used to compute a reproduction d' to establish whether a participant had no experience of the stimulus (d' = 0); and how the graded character of reproduction responses allow for more nuanced analysis in which the graded strength of the subjective experience can be related to trial-level neural measures (e.g. EEG).

2. Second, it is unclear whether reproduction responses (even in the control condition) are also biased in trials where participants report "present," potentially leading to systematic overestimations of the reproduced feature.

Here, we were not entirely sure whether 'where participants report "present,"' refers to (i) an explicit 'present' response (as in the detection task) or to (ii) trials in which participants produced a *non-zero* reproduction (i.e. a present reproduction). To be sure, we address both possibilities.

(i) In Experiments 1 and 2, participants provided only one response type on each trial, that is, either a present/absent response or a reproduction of the contrast of the patch. In Experiment 3, participants gave a reproduction response on every trial. Thus, in reproduction trials, there was no accompanying present/absent response. Because there is only one response on every trial (either detection or reproduction) we cannot know whether participants would be more biased on reproduction trials in which they also report presence; we can only assess this at a block level. We now make this explicit in lines 123-124.

(ii) We next consider whether trials in which participants gave a *non-zero* reproduction may have been biased even in the control condition. The main confound we find in the reproduction paradigm is that the decision to reproduce can be contaminated when the reproduction task is embedded in a detection context, as shown in Experiment 1 and 2 (e.g. Figure 3F, 3G, 4F and 4G). We directly addressed this issue in Experiment 3, in which we showed that removing the detection task eliminated this decisional contamination. Thus, removing the decision context also removes any non-perceptual overestimation of the reproduced feature, as can be seen in the base-rate figures of Figure 5. However, Figure 5 also shows that there is still a slight non-zero contrast reproduction even when the true contrast is zero (absent trials) in the control condition for both the attentional cue and the base-rate condition. This means that indeed, reproduced contrast can be slightly overestimated even when the true contrast is zero (i.e. a 'false alarm'), even in the control condition. It is not clear whether this should be characterized as a 'bias' or a true perceptual effect. This effect could be due to the asymmetry of the reproduction response (one can only reproduce zero or positive contrast values but not negative contrast values), so that any noisy response pattern is likely to always be slightly positive. Regardless, any non-perceptual bias contribution to reproduction responses should be unspecific and in principle present both in the control and in the biased condition, as we also show here. Thus, this difference should be cancelled out when comparing the control and biased condition, as we do in our study.

3. Third, I found the term "detection threshold shift" a bit misleading when referring to a post-perceptual bias rather than a genuine change in sensitivity.

We thank the reviewer for pointing out this potential ambiguity. We most commonly used the term "detection threshold shift" to refer to a change in the decision criterion used by participants in the detection task. Note that such a criterion shift in the detection task does not necessarily imply a change in sensitivity but rather reflects a change in the propensity to indicate presence or absence as reflected in the proportion of absent responses. However, we now realize that the term "detection threshold shift" was ambiguous and could be interpreted as a change in the detectability of the stimuli, and therefore as a change in sensitivity instead of a post-perceptual effect. Further, note that we relate this criterion shift in the detection task to a change in the threshold that determines whether to reproduce or not in the reproduction task, based on the strong correspondence between the proportion of absent responses in the detection task and the model-predicted proportion of *zero* reproductions in the reproduction task (Figures 3F and 3G, as well as 4F and 4G). As a result, we also sometimes used the term "detection threshold" in relation to the reproduction threshold, potentially leading to further confusion. We have made several changes to remedy these problems. First, we have replaced the term "detection threshold shift" with "decision criterion shift" and the

term “detection threshold” with “decision criterion in the detection task” across many parts of the manuscript where relevant (too many lines to indicate here; see the highlights in the tracked_changes.pdf document). Further, we realized that it was ambiguous to sometimes use the term “detection threshold” in relation to the threshold in the reproduction task. Where relevant, we now consistently refer to the threshold in the reproduction task as the reproduction hurdle (changed made across too many lines to mention here) to differentiate from the decision threshold in the detection task. We hope this clarifies our usage of these terms and resolves any ambiguity that may have existed.

4. In the introduction, reproduction tasks are presented as the better method, but the rationale for this claim is not well developed. Reproduction tasks are themselves susceptible to multiple types of biases, so it would be important to explain why, in this specific experimental context, they might nonetheless be preferred—ideally already in the opening of the paper.

This is an important point, and we thank the reviewer for this suggestion. In the revised manuscript, we now explain the rationale for using the reproduction task (lines 79-86). Importantly, we clarify why the reproduction task is particularly suitable in the present context: unlike explicit detection reports, the reproduction approach does not require participants to adopt a decision criterion to distinguish between present and absent trials, thereby avoiding criterion-related decision biases. Indeed, in our previous work, we have shown that reproduction measures were able to clearly distinguish between manipulations that influence perception and those that affect decision-making. We also make explicit that we cannot be sure that biases due to history and/or contextual effects play a role in reproduction of contrast but explain that biases in reproduction are generally considered to be perceptual in nature (lines 87-96). This supports rather than detracts from reproduction as a valid measure of conscious experience. Finally, we point out that although the motor system can cause biases in reproduction, we address this issue in Experiment 2 and 3.

5. Finally, I wonder whether a factorial analytical approach (e.g., ANOVA with main effects and interactions) might have been more appropriate than multiple pairwise t-tests.

The main goal of our study was to investigate to what extent present/absent detection judgments are affected by perceptual and/or non-perceptual biases. However, we did not know beforehand whether the effects we investigate would manifest as main effects across contrast levels or whether they would manifest specifically on present or absent trials. Therefore, we decided it was more appropriate to perform three planned comparisons per bias type (attentional cue, base rate and payoff) and task, that is, to test whether there was a difference between the biased and the control conditions, separately at each contrast level (absent, low and high), in both the detection and reproduction task. Thus, although we could in principle perform a repeated-measures ANOVA with contrast level and condition as factors, this would still require pairwise comparisons to determine whether a given manipulation increased present responses (detection task) and reproduced contrast (reproduction task) for absent, low and high contrast trials. Note also that we perform a rather reduced number of planned comparisons (three) compared to the potential fifteen pairwise comparisons that can be performed in a 2 x 3 design. Because of this reason, we believe the current approach is more suitable than a factorial analysis approach.

Minor:

Figure 2, panel C: “contrast” is misspelled as “conctrast.”

Thank you for pointing this out. We have corrected the figures.

Reviewer #2 (Remarks to the Author):

In this manuscript, the authors aim to disentangle perceptual from decisional factors by comparing how contextual factors (attentional cues, base rates, and payoffs) impact performance and bias using two tasks: a detection task (target present/absent) and a reproduction task (reporting perceived contrast.) Behavioral results and Hurdle–Gaussian modeling suggest that attentional cues modulated perception, and base-rate and payoff manipulations primarily induced decision biases that also affect reproduction results. The authors suggest that stimulus detection influences reports in reproduction tasks, and that reproduction tasks should be preferred to detection tasks when measuring conscious perception. The manuscript is well written, and the experiments, modelling and logic of the approach are generally clear. However, there are several issues that should be addressed to improve the manuscript.

1. The manuscript falls between a rather narrow focus, centered on a specific combination of a detection and reproduction task, and much broader theoretical claims regarding the distinction between perceptual and decisional processes. It would be good to better define the intended scope of the current findings. While the title suggests a relatively specific topic, the broader goal seems to imply implications for the study of perception and bias in general.

Author responses in green

We want to first thank Reviewer #2 for taking the time to read our manuscript and for the helpful comments provided. We believe the revised document is a better manuscript because of them.

Indeed, the broader goal of our study was to disentangle perception and bias in detection tasks (so at the intersection of a broader theoretical goal to separate perceptual from bias effects, but with a specific application in consciousness research that typically uses detection to measure consciousness). The findings therefore imply a broad biasing effect when using detection tasks, either stand-alone or together with other measures of consciousness. This is what we tried to convey in the original title, but we agree the title might be improved to better reflect the main aim and conclusion. We have now revised the title to: "Disentangling perceptual and response biases: The act of detecting a stimulus contaminates measures of conscious experience". We have now also added and/or rephrased parts of the introduction to better reflect this broader goal (see line number 105-108 in tracked_changes.pdf).

2. Whether the approach successfully dissociates perception and decision bias is not clear. While there is strong evidence for changes in criterion with attentional cues, base rate manipulations, and different payoffs, and many studies have shown that attention may change appearance, whether perceived contrast actually changed in the attention condition remains unclear. In this context, it would be good to discuss studies that suggest that perceived contrast is not changed by attention (for example, Schneider, 2006, 2011, 2021; Schneider & Komlos, 2008).

We thank the reviewer for pointing out this important body of work. We have revised the Discussion to consider these alternative accounts that argue that exogenous attentional cueing does not alter perceived contrast but instead produces decision bias (lines 615-628). Note that prior to collecting our data we had also been somewhat sceptical that the Carrasco's attentional cueing result was due to a shift in appearance and could not be explained by a decisional bias. We would therefore like to emphasize that our paradigm avoids the comparative and equality judgements often

criticised for decision contamination. Instead, we combine a detection and reproduction task to provide an independent benchmark of subjective experience. With this approach, we find that exogenous cues not only increase detection reports but also shift the average reproduced contrast (figures 2D and 4B), consistent with a perceptual change. Further, our results show that this shift in the average reproduced contrast only happens in the attentional cue condition and does not occur in the other biased conditions (payoff and base rate), which only see a shift in the number of non-zero reproductions. Although we believe this provides compelling evidence, it is important to highlight that it is intrinsically impossible to establish with definitive certainty whether any given measure, including the reproduction task, uniquely assays subjective experience. However, based on the result from our previous studies using the reproduction metric (Sánchez-Fuenzalida et al., 2023; 2025) in combination with the extensive experimental and computational work we carried in the current study, we cannot identify any further confound that could be driving our results. Therefore, we believe our conclusion, that the reproduction task distinguishes between perceptual and decision biases, is well-grounded.

3. Regarding the difference between the attentional cue and the other two conditions in Experiments 1 and 2: do we know whether the change in decision criterion influenced reports in the reproduction task or is the interpretation of these results only meaningful when also considering the results of the control experiment?

Our interpretation that a change in decision criterion influenced reports in the reproduction task is based on the close similarity between the model-predicted proportion of *zero* reproductions and the proportion of "absent" responses in the detection task in Experiments 1 and 2 (see Figures 3F and 4F). This parallel strongly suggests that the decision criterion in the detection task also determined the rate of zero and non-zero responses in the reproduction task. Although compelling, without Experiment 3 this explanation might still be considered somewhat speculative. The results of Experiment 3 further confirm that when the detection task was removed, the base rate effect in reproduction disappeared as well, whereas the effect of the attentional cue remained. We have made this line of reasoning more explicit (lines 517-523 and 561-570). Note also that in future research, the reproduction task should be used alone, as in Experiment 3, so that there would not be a decision criterion effect to influence reports in the reproduction responses.

4. Related to that, to what extent were participants aware of their false alarms/ liberal criteria, and what is the influence of that on zero/non-zero responses? Confidence ratings and reaction times could be helpful in this context as well.

We cannot know for sure whether participants were aware of their decision bias because we did not measure this. However, in Experiments 1 and 2 participants were aware of the advantages of adopting a liberal criterion (higher expected rewards in the payoff condition and higher probability of being correct in the base rate condition), therefore we are inclined to think they were at least partially aware of their liberal criterion. Based on the same reasoning, we would be inclined to say participants were not aware their detection decision criterion was affecting their reproduction responses, as there was no obvious advantage of adopting a liberal criterion in the reproduction task.

Although using confidence reports is an interesting suggestion, we want to highlight that confidence reports may not be able to fully dissociate perceptual and non-perceptual effects. We have shown before (Sánchez-Fuenzalida et al., 2025) that non-perceptual decision biases caused by

manipulations similar to the ones we employ in this study (base rate and payoff) leak to confidence reports in a discrimination setup. We now discuss the use of confidence reports and metacognition as well as other methods to study conscious perception in lines 689-725. In short: participants use information that is relevant for their performance to inform their confidence reports (base rate), but also information that has no bearing on their performance, such as the expected rewards associated with a choice (payoff).

Further, in the current setup we cannot perform analyses on the reaction times of either of the tasks because the delay between the target offset and the start of window response removes any differential signature reaction times could carry. It is likely that RT effects on the reproduction data would be less reliable even without the delay, because the reproduction response is a more drawn-out response in which the participant needs to continually change the contrast of the item on the screen to match it with what they had just observed. Thus, taking the moment at which they first touch the scroll wheel as RT is dissimilar from a categorical decision, which starts and ends with a single button press, because participants know they have more time to finalize their decision even after touching the scroll wheel for the first time. In a prior article in which we introduced the decision plus reproduction approach we did look at RT data for the decision response alone. For this we had to employ a different sample to model reaction times using Drift Diffusion Modelling on the decision responses (Sánchez-Fuenzalida et al., 2023). The key difference in that sample was that participants only performed a discrimination decision and there was no delay between the target and the response window, so that we could use RTs to run the DDM model. Unfortunately, this delay is one of the limitations of the reproduction approach, as it renders reaction time data uninformative.

Sánchez-Fuenzalida, N., Van Gaal, S., Fleming, S. M., Haaf, J. M., & Fahrenfort, J. J. (2023). Predictions and rewards affect decision-making but not subjective experience. *Proceedings of the National Academy of Sciences*, *120*(44), e2220749120.

Sánchez-Fuenzalida, N, van Gaal, S., Fleming, S. M., Haaf, J. M., & Fahrenfort, J. J. (2025). Confidence Reports during Perceptual Decision Making Dissociate from Changes in Subjective Experience. *Communications Psychology* 3, no. 1: 1–11.

1, 2, 3, 4) These points could be discussed in more detail in the General Discussion, possibly discussing a broader perspective on methodological alternatives (e.g., two-alternative forced choice tasks, confidence reports etc.), and implications for consciousness research.

We have answered points 1 through 4 individually where they occur. It is a good suggestion to also discuss alternative methods in the discussion. We have therefore also added a section to the Discussion (lines 685-725) in which we outline alternative methodologies along with their advantages and limitations.

5. A key contribution of this study seems to be to disentangle perceptual effects from decisional biases. However, that is not clear throughout the paper, including the abstract concluding with “We propose that reproduction is better suited to assay conscious experience than detection” and the general discussion. It could be stated more explicitly how this approach can disentangle the two beyond the paradigm used here. Specifically, how applicable are these findings for studies of conscious perception more broadly and how

successful is the model in determining whether a stimulus was consciously experienced, especially in more ambiguous cases?

We have now included a section in the Discussion (lines 658-684) in which we outline how the reproduction approach can be used more widely in consciousness research. In short, reproduction responses reflect the subjective strength of the perceptual experience: non-zero reproductions indicate some perceptual experience, whereas zero reproductions reflect its absence. We also highlight how this approach can be combined with manipulations of awareness (masking, attentional blink, continuous flash suppression, etc.) to study the difference in the processes that underlie consciously perceived stimuli and stimuli that remains unconscious. Note that the hurdle Gaussian model is specifically useful when reproduction is combined with detection, because in that situation reproductions can have two underlying causes: a decision component and a reproduction component. The decision component can be dissociated from the reproduction component using the model. However, because detection intrinsically introduces decisional bias, we argue that it may be better to not use detection tasks and simply replace them with reproduction.

6. It seems that Experiment 1 had initially a different goal, as the authors write "However, we realized that an increase in the average reproduced contrast as reported in Figure 1D may in fact result from two distinct influences on reproduction behaviour: on the one hand, the overall average reproduced contrast reflects the reproduced contrast on non-zero contrast reproductions, but on the other hand, it also reflects the ratio of zero to non-zero contrast reproductions." Given that several issues in Experiment 1 were addressed in Experiment 2, it might have been clearer to present Experiment 2 as the main experiment—based on the exploratory analysis of Experiment 1, especially considering the rather detailed description of Experiment 1.

We thank the reviewer for pointing this out. However, we want to clarify that Experiment 1 always had the same goal, that is, to establish which of the three bias manipulations (base rate, payoff and attentional cue) had an effect on perception and which had an effect on decision bias. What we had not anticipated was that the reproduction responses would be affected by the decision criterion that observers used in the detection task. We did not anticipate this possibility because in previous work, the reproduction task was not affected by the decision context in which it was embedded (Sánchez-Fuenzalida et al., 2023, 2025), plausibly because those experiments involved discrimination rather than detection. To overcome this problem, we devised the Hurdle-Gaussian analysis framework to effectively separate the contributions of a shift in detection criterion and perceived contrast on reproduction responses. Because these analyses were necessarily exploratory, we designed Experiment 2 as a direct replication with improved methodology. Importantly retaining Experiment 1 allows us to demonstrate that Experiment 2 constitutes a direct replication of the initial findings, thereby providing stronger grounds for the robustness of our conclusions. To minimize the overhead involved in describing the same experiment twice, in the main text and in the Methods we highlight only the methodological changes we made in Experiment 2 when compared to Experiment 1.

Sánchez-Fuenzalida, N., Van Gaal, S., Fleming, S. M., Haaf, J. M., & Fahrenfort, J. J. (2023). Predictions and rewards affect decision-making but not subjective experience. *Proceedings of the National Academy of Sciences*, *120*(44), e2220749120.

Sánchez-Fuenzalida, N, van Gaal, S., Fleming, S. M., Haaf, J. M., & Fahrenfort, J. J. (2025). Confidence Reports during Perceptual Decision Making Dissociate from Changes in Subjective Experience. *Communications Psychology* 3, no. 1: 1–11.

7. While most parts are explained well in high detail, certain parts remain unclear.

For example, in the results section, the authors write: "Each of these conditions which was accompanied by a neutral control condition (see Figure 1B for a graphical summary of the manipulations)." However, Figure 1B does not clearly explain what the neutral condition exactly is.

Thank you for highlighting this issue. We realized we had inadvertently not used consistent condition labelling across the figures / figure panels. The control condition that contained No bias manipulation was called “No bias” in Figure 1B, but “Control” in other panels. We have now consistently labelled the neutral condition as ‘Control’ in Figure 1B and all other relevant panels to better reflect the text “Each of these three bias conditions was accompanied by a within-subjects neutral control condition in which no bias manipulation was implemented (see Figure 1B for a graphical summary of the control and bias conditions).” (lines 132-133, 137-138). We have also checked all text to make the labelling consistent throughout. We hope these modifications resolve any misunderstandings that may have occurred.

8. It could be said more explicitly how the control experiment shows the following: "Crucially, this control experiment shows that the effect the base rate had on the proportion of zero reproductions in experiments 1 and 2 disappeared when removing the detection context during reproduction, clearly favouring the interpretation that attentional cues influence subjectively perceived contrast (conscious perception), while the effect of base rate is non-perceptual in nature."

We have revised this paragraph to make our line of reasoning more explicit (lines 561-570). In short, because the key difference between Exp. 2 and 3 is the detection context in which the reproduction task is embedded in Exp. 2, we conclude that the shift in the proportion of zero reproductions in the base rate condition must be caused by detection threshold adopted in the detection task leaking into reproduction responses. The alternative explanation is that the shift in zero reproductions in the base rate condition was caused by the higher stimulus prevalence, reflecting stimuli being consciously perceived more often. However, the physical stimulation was identical in Exp. 2 and 3, meaning that we should have seen the effect of base rate on the proportion of zero reproductions in Exp. 3 if that would be the case. In contrast, the attentional cue continues to influence all the features of reproduction responses (overall reproductions, proportion of zero reproductions and distribution of non-zero reproductions). Together, these results led us to conclude that the effect of the attentional cue must be perceptual whereas the effect of base rate must be decisional.

9. What type of "different" pattern was expected? "If the detection task had not influenced how participants behaved in the reproduction task, the pattern of results should not be affected when removing it. However, if the detection task inadvertently affected how participants respond in the reproduction task, the reproduction task without detection would be expected to show a different pattern of results despite identical physical stimulation."

We have revised this paragraph (lines 960-962) to better explain what we mean:
“We reasoned that if the detection task had not influenced how participants behaved in the reproduction task, the pattern of results should be the same as in experiment 1 and 2 when removing it. However, if the detection task inadvertently affected how participants respond in the reproduction task, the reproduction task without detection would be expected to show a different

pattern of results compared to Experiments 1 and 2, despite identical physical stimulation. Specifically, we would expect the effect of the bias manipulation on the proportion of zero reproductions to disappear in Experiment 3, if it was artificially induced by the detection requirements present in Experiments 1 and 2."

10. Explicitly mention why there was a big difference in the number of participants: "In experiment 3 we aimed to collect the same number of participants as in experiment 2 after removing Outliers."

We are not quite sure which difference the reviewer is referring to here because Experiment 2 and Experiment 3 had identical participant numbers (see table below). Maybe the reviewer means the difference in the number of participants between experiment 1 and 2? To be sure, we explain the sample sizes in more detail below. In Experiment 1, we employed an optional stopping rule to determine the total sample size. The stopping rule consisted of having moderate evidence (BF >= 3 or BF <= 0.33) for the comparison of control and bias conditions in absent, low and high contrast trials in the detection task (see Figure 1C) and in the reproduction task (see Figure 1D). That is, for each bias type (attentional cue, base rate and payoff), all these six comparisons had to yield at least moderate evidence for an effect (BF >= 3) or for the null (BF <= 0.33) to stop data collection. The attentional cue required a larger number of participants mainly due to the comparison of reproduction responses between the control and bias condition in absent trials. There, the effect size was small and required a larger number of participants to achieve moderate evidence. In Experiment 2, to prevent collecting such many participants, we performed a sequential sampling plan based on the effect sizes of Exp. 1. The sequential sampling plan indicated that 85 participants would yield a power of 90%. Therefore, we collected the data of at least 50 subjects and continued until had moderate evidence on the main comparisons (same as in Exp. 1; see Figure S3A and S3B) or until we collected 85 participants. Finally, in Experiment 3 we aimed to collect the same number of participants as in Experiment 2.

Note that the biggest difference in participant number takes place in Experiment 1, where the attentional cue condition has roughly double the participants compared to the base rate and payoff condition. We do not know why the attentional cue required much more participants in Exp. 1 but not in Exp. 2 or 3. One potential reason is that the changes we made to the way participants reproduced in Exp. 2 and in Exp. 3 (randomly changing the scrolling direction on every trial) resulted in a cleaner effect and therefore in fewer participants needed. In any case, given that we replicated the results of Experiment 1 in Experiment 2, in which the participant counts are similar across conditions (only 8 participants more in the base rate condition), this difference in participant counts is not problematic. Also note that in Experiment 3, again the difference between the attentional cue and the base rate condition is only 8 participants. We now explicitly mention the reason for the difference in the number of participants (lines 759-766).

**Exp. \ Condition**	**Att. Cue**	**Base rate**	**Payoff**
**1**	105	57	64
**2**	50	58	50
**3**	50	58	

11. When d′ is mentioned, the curly quotation mark appears to be used instead of the straight apostrophe.

Thanks for pointing this out, we have now replaced the curly quotation marks with straight apostrophes throughout the text.

12. The legends of the figures could be improved.

We have revised the legend of the figures to improve clarity and to make them more self-contained.

13. Introduce and define clearly terms that might not be familiar to the average readers of the journal, for example, define zero and non-zero reproductions; occasional dense passages may lose readers less familiar with the topic.

We have better explained what we mean by zero and non-zero reproductions (lines 207-214) and we have reworked multiples passages to improve clarity.

Reviewer #3 (Remarks to the Author):

The study “The act of detecting a stimulus contaminates measures of conscious experience with unrelated cognitive factors” seeks to distinguish perceptual from decision effects in consciousness judgments. Participants viewed briefly presented with Gabor patches that varied in contrast (low, high) across trials. In experiments 1 and 2, participants were asked to perform either a detection task (present or absent) or a reproduction task (adjusting the contrast of the Gabor to match their perception). Importantly, the study introduced three types of bias (manipulated between participants), a base rate bias, a payoff bias, and an attentional cue bias. The results of the first two experiments showed that although all bias conditions elicited more “present” responses in the detection task, only the attentional cue condition resulted in an increase in perceived contrast in the reproduction task for detected targets. The authors conclude that the base rate and payoff conditions induced decision bias whereas the attentional cue resulted in an actual perceptual shift. Modeling the data using a Hurdle-Gaussian model supported the presence of both types of bias in the data. A control experiment suggested that detection tasks may induce decision bias, therefore contaminating measures of conscious experience.

The method of the study is novel and the results very interesting, making a contribution to the ongoing debate about how to best measure conscious experience. I have a few comments about the methodology and interpretation of the results.

1. To me the results show a clear perceptual effect in the attentional cue condition. However, it is still worth thinking whether the results in the base rate and payoff conditions are purely decision-based.

   If bias is purely decision based, this should be reflected in the non-zero contrast reproduction performance across contrasts. Let’s take the base rate condition for example. We expect that in the control condition, there would be a difference between reproduced contrast in the high compared to the low contrast condition because these judgments largely contain truly perceived trials. However, in the bias condition, the difference between low and high contrast conditions should be affected by not only truly perceived trials (which differed in perceived contrast), but also trials in which participants responded without having any true perceptual experience (which presumably elicited similar reproduced contrast across contrast conditions because nothing was perceived). In other words, in the control condition the comparison between high and low contrast reflects “signal” while in the biased condition the same comparison reflects “signal” plus some “noise” which should be of similar magnitude across contrast levels. I wonder if the authors could address whether the difference between high and low contrast conditions are consistent with the idea of “noise” (which would be consistent with decision bias) or not (which would be consistent with a perceptual account).

We thank the reviewer for this thoughtful suggestion. However, how the proposed analysis provides additional information about the perceptual nature of the base rate and payoff effects is not so clear to us. As the reviewer correctly points out, the additional bias (what the reviewer calls “noise”) should be of approximately similar magnitude across contrast levels. Comparing high to low contrast would not help to identify such bias, because both the high and the low contrast condition would be approximately equally affected by the “noise” (bias) and would thus be subtracted out when comparing low and high contrast. What the reviewer seems to want us to inspect is if the

increase in average reproduced contrast (based on the non-zero contrast reproductions) between low and high contrast is the same for the control condition when compared to the bias condition, so that if you would see a difference there (e.g. a larger difference between high and low in the bias condition than in the control condition), this might point to a perceptual effect in the bias condition. However, this difference is already isolated by making comparisons within contrast levels, because if such a difference would exist, it would appear there too.

To illustrate, let us denote the non-zero reproduction effects as follows: reproduction of low contrast in the control condition is A, reproduction of high contrast in the control condition is B, reproduction of low contrast in the bias condition is C, and reproduction of high contrast in the bias condition is D. What the reviewer seems to want to inspect is this:

B – A = perceptual_effect_control_condition

D – C = perceptual_effect_bias_condition

and then to see whether perceptual_effect_bias_condition > perceptual_effect_control_condition?

However, we already show that A – C = 0 and B – D = 0 (in Figures 2D, 4B and 5C). In these figures one can see that for the base rate and payoff conditions, *non-zero* reproductions are the same between the control and biased condition for both contrast levels (i.e. low and high contrast). This already rules out the possibility that (B – A) will produce a different result from (D – C) because these operations are commutative. If (B – D) = 0 and (A – C) = 0, then in fact B = D, and A = C, which means that (B – A) must also be equal to (D – C). Therefore, there is nothing to be gained from this analysis over and above what we have already shown. We hope this clarifies. If not, we are happy to perform more control analyses, but it is currently not clear to us how the proposed analysis adds to what we have already shown.

2. The proposed two-step model, binary (threshold) followed by a linear (contrast) judgments, makes predictions about reaction time. For example, as I understand it, RT for zero contrast judgments would be faster because on these trials processing end after the first step. There are of course more predictions one can make. It would be interesting to look at RT results and also investigate differences in Experiment 3 in which target processing presumably changes in the absence of the detection task.

We thank the reviewer for bringing up this interesting idea. However, to make sure observers do not start reproduction before they have accumulated all evidence, there is a delay between the offset of the target patch and the start of the response window (600, 700 or 800 ms in Experiment 1, or fixed at 600 ms in Experiments 2 and 3). Unfortunately, this delay removes any differential signature reaction times may carry. Furthermore, it is likely that RT effects on the reproduction data would be less reliable even without the delay, because the reproduction response is a more drawn-out response in which the participant needs to continually change the contrast of the item on the screen to match it with what they had just observed. Thus, taking the moment at which they first touch the scroll wheel as RT is different from a categorical decision, which starts and ends with a single button press, because participants know they have more time to finalize their decision even after touching the scroll wheel for the first time. In a prior article in which we introduced the decision plus reproduction approach we did look at RT data for the decision response alone. For this we had to employ a different sample to perform Drift Diffusion Modelling on the decision responses (Sánchez-Fuenzalida et al., 2023). The key difference in that sample was that participants only

performed a discrimination decision and there was no delay between the target and the response window, so that we could use RTs to run the DDM model. Unfortunately, this delay is one of the limitations of the reproduction approach, as it renders reaction time data uninformative.

Sánchez-Fuenzalida, N., Van Gaal, S., Fleming, S. M., Haaf, J. M., & Fahrenfort, J. J. (2023). Predictions and rewards affect decision-making but not subjective experience. *Proceedings of the National Academy of Sciences*, *120*(44), e2220749120.

3. The title could be improved. Unrelated cognitive factors sounds a bit too general. The title seems to disproportionately emphasize experiment 3 rather than represent the entire study.

Thank for pointing this out. Motivated by this comment and other comments by Reviewer# 2, we have modified the title to better represent the entire study. The new title reads "Disentangling perceptual and response biases: The act of detecting a stimulus contaminates measures of conscious experience" to make explicit that the confounding effects are caused by non-perceptual biases.

4. The fact that the tasks were manipulated between subjects was not immediately clear to me. I eventually found it in the methods. It would be good to clarify earlier/more clearly in the manuscript and figure. The fact that the N is so uneven between conditions (and much higher for the condition that shows the perceptual effect in Experiment 1) also raises a flag and should be addressed.

Thank you for pointing this out. We have modified the text in lines 132-133 and 137-138 in tracked_changes.pdf, and Figure 1 to explicitly indicate that the attentional cue, base rate and payoff were between-subjects manipulations.

Regarding the difference in participant counts, the attentional cue, base rate and payoff conditions have different participants counts because we apply an optional stopping rule to determine when to stop data collection. The stopping rule consisted of having moderate evidence (BF >= 3 or BF <= 0.33) for the comparison of control and bias conditions in absent, low and high contrast trials in the detection task (see Figure 1C) and in the reproduction task (see Figure 1D). That is, for each bias type (attentional cue, base rate and payoff), all these six comparisons had to yield at least moderate evidence for an effect (BF >= 3) or for the null (BF <= 0.33) to stop data collection. We also want to note that biggest difference takes place in Experiment 1, where the attentional cue condition has roughly double the participants compared to the base rate and payoff conditions. The attentional cue in Experiment 1 required a larger number of participants mainly due to the comparison of reproduction responses between the control and bias condition in absent trials. There the effect size was small and required a larger number of participants to achieve moderate evidence. We do not know why the attentional cue required much more participants in Exp. 1 but not in Exp. 2 or 3. One potential reason is that the changes we made to the way participants reproduced in Exp. 2 and in Exp. 3 (randomly changing the scrolling direction on every trial) resulted in a cleaner effect and therefore in fewer participants needed. In any case, given that we replicated the results of Experiment 1 in Experiment 2, in which the participant counts are similar across conditions (only 8 participants more in the base rate condition), we do not think this difference in participant counts is problematic. Also note that in Experiment 3, again the difference between the attentional cue and the base rate condition is only 8 participants. We now explicitly mention the reason for the difference in the number of participants (lines 759-766).

**Exp. \ Condition**	**Att. Cue**	**Base rate**	**Payoff**
**1**	105	57	64
**2**	50	58	50
**3**	50	58	

5. Since the authors emphasize the results of experiment 3, this experiment should have its own clearly marked section. It would be helpful to include more discussion on how the absence of a detection task changes the processing of the stimulus (replacing the two step model) and how zero-contrast judgments may arise from qualitatively different processes compared to "absent" responses in the detection task.

Thank you for these suggestions. We have now better delineated and appended a shorthand title above the section of each experiment, including Experiment 3:
Experiment 1: reproduction and detection
Experiment 2: replication and modelling to disentangle sources of bias
Experiment 3: reproduction without detection

We have also added a section to the Discussion (lines 643-657) in which we discuss how the presence and removal of the detection ask may have changed reproduction responses to the stimulus. In short, we explain now that the processing of the stimulus is not different when there is a detection context that accompanies the reproduction task. Instead, the detection context adds a decision layer that operates on the already perceived stimulus. We also explain how this decision layer makes "absent" detection responses different from *zero* reproductions.

6. Line 496: cued trials resulted in a higher proportion of zero reproductions across all contrast levels" should that be "lower", or am I misunderstanding?

Indeed, this was a typo and should have been "lower". Thank you for pointing this out.

Reviewer #3 (Remarks on code availability):

I did not try to run the code but I can access the Github with all the files.

Dear Editor,

Thank you for the opportunity to revise our manuscript. We have carefully revised the manuscript according to the comments and suggestions provided by the reviewers. We believe we have incorporated all the suggestions into the manuscript.

Below we provide a document with detailed point-by-point responses to reviewers' comments referring to the locations in the manuscript where changes have been made. For convenience we also provide a document that highlights all the changes that were made relative to the original submission (tracked_changes.pdf).

Thank you for your time and consideration, we hope the revision now meets the requirements for publication in Nature Communications.

Sincerely,

Nicolás Sánchez-Fuenzalida
Chris Jungerius
Stephen M. Fleming
Simon van Gaal
Johannes J. Fahrenfort

Reviewer comments

Reviewer #1 (Remarks to the Author):

The authors have addressed my main concerns, but one part added in response to my comments remains misleading. Specifically, the manuscript now suggests that attractive serial dependence, one of the classic sources of bias in reproduction tasks (and also in forced-choice tasks), is purely perceptual. The current state of the literature points in the opposite direction: attractive history biases are generally understood to arise largely, if not predominantly, at post-perceptual stages. More appropriate references for reviews and key findings include (Barbosa et al., 2020; Ceylan et al., 2021; Fischer et al., 2024; Fritsche et al., 2017; Luo et al., 2025; Pascucci et al., 2019, 2023; Sadil et al., 2023; Sheehan & Serences, 2022) (to mention but a few). Of course I am not requesting to cite all these references, just to give a better sense of the current state of knowledge and I therefore strongly encourage the authors to frame this section in accordance with what is actually known from the broader empirical literature rather than relying on a single opinion.

In addition, history-dependent biases are not the only sources of systematic errors in reproduction responses. Well-documented effects such as regression to the mean and stimulus-specific biases, whose expression depends on the structure of the stimulus space (e.g., linear, constrained, circular), also contribute, and these too are likely to involve post-perceptual processing (see also 'swap errors' in the domain of working memory research).

Related to this, the conclusion that reproduction tasks are sensitive to perceptual manipulations but not to post-perceptual ones is misleading. Several studies, for instance, show clear influences of post-perceptual manipulations such as memory load and feedback on reproduction performance and biases (Balikou et al., 2015; Markov et al., 2024).

For these reasons, if, in addition to the argument of avoiding a binary threshold, the authors' motivation for preferring reproduction responses over forced-choice judgments is based on a presumed selective targeting of perceptual rather than post-perceptual processes, I find this argument unconvincing and potentially misleading.

Author response in green

We thank the reviewer for this detailed and constructive comment. We agree that our earlier wording could be read as suggesting that serial dependence is "purely perceptual" and that reproduction tasks are selectively sensitive to perceptual (but not post-perceptual) processes. This was not our intention, and we appreciate the opportunity to clarify and nuance this part of the manuscript.

Our aim was to argue for the perceptual basis of reproduction tasks, including some perceptual biases, not that they are exclusively perceptual. Indeed, we ourselves show in the manuscript that reproduction is also sensitive to non-perceptual biases when a reproduction task is randomly intermixed with a detection task, so this claim would have contradicted our own main finding. We fully agree with the reviewer that the broader literature on reproduction converges on a more complex picture, with both perceptual (e.g. due to adaptation) and post-perceptual contributions (e.g., from working memory, decision, or response stages).

In the revised manuscript, we have now removed any phrasing that might have suggested that reproduction biases such as serial dependence are “purely” or “uniquely” perceptual (lines 102-120). We explicitly acknowledge the contribution of both repulsive and attractive dependencies in reproduction of orientation, and that the latter are generally understood to involve post-perceptual components, as emphasized by recent work (e.g. Ceylan et al., 2021; Fischer et al., 2024; Fritsche et al., 2017; Luo et al., 2025; Pascucci et al., 2019, 2023; Sadil et al., 2023; Sheehan & Serences, 2022). We further clarify that the evidence we cited (e.g. Fritsche et al., 2017; Fritsche & Lange, 2019; Cicchini et al., 2024) speaks to the existence of perceptual contributions to serial repulsive effects, but that these do not rule out the presence of simultaneous post-perceptual influences.

Furthermore, we agree that history-dependent biases are not the only sources of systematic error in reproduction paradigms. In the revised manuscript we make explicit that there are also other post-perceptual influences on reproduction, such as caused by performance feedback, and motor repetition, some of which we explicitly addressed in Experiment 2. We discuss this more elaborately when we introduce Experiment 2.

However, we also point out that the existence of post-perceptual serial attraction or other non-perceptual phenomena do not undermine our main conclusions about the influence of detection related criterion biases when attempting to measure conscious experience. Our main argument for using reproduction over binary detection is not that reproduction taps only perceptual processes (although perceptual contributions do dominate over non-perceptual ones in reproduction) but that it avoids the need to set an explicit presence/absence criterion, making it less directly exposed to criterion placement confounds that are central to yes/no detection in consciousness research (lines 82-91). In both this paradigm and our prior work (Sánchez-Fuenzalida et al., 2023, 2025), we use reproduction to dissociate perceptual vs. non perceptual criterion effects through careful experimental design in which different criterion manipulations are compared, and combined with computational modelling to test auxiliary hypotheses, rather than claiming a general selective sensitivity of reproduction to perception only. The removal of the detection task in Experiment 3 clarifies that detection itself is a particularly strong source of non-perceptual effects on reproduction responses.

The main rationale for the reproduction task and potential non-perceptual influences on reproduction are now discussed in much more detail in the Introduction, the Discussion, as well as in the section that introduces Experiment 2 (lines 445-468). We hope these changes address the reviewer’s concern and prevent any misleading interpretation of our claims.

Reviewer #2 (Remarks to the Author):

With the changes and additions, the manuscript has improved overall. While the revised manuscript discusses potential broader implications of the results, the scope remains somewhat vague. It would strengthen the manuscript to explicitly define the scope earlier in the text, clarifying to what extent the findings go beyond contrast detection or discrimination with simple Gabor stimuli and the parameters chosen here. This should include a discussion of the limitations due to the types of stimuli suitable

for reproduction tasks, while also acknowledging that reproduction tasks, and more generally appearance-based tasks, are already common in the consciousness literature. Avoiding potential overgeneralization when interpreting the broader applicability of the results seems important.

Author responses in green

We thank the reviewer for the additional constructive comments. In the revised manuscript, we now explicitly define the scope at the end of the Introduction (lines 144-153) clarifying that our main empirical results involve contrast-defined Gabor stimuli, and a reproduction task in a low-dimensional stimulus space (i.e. contrast). However, we also point out that our results bring something general to bear on the consciousness literature. Presence/absence judgements are central to consciousness research. A Google Scholar search for the keywords "detection task" AND consciousness yields a whopping 23.000 results, while a search for the keywords (“reproduction task” OR “appearance task” OR “adjustment task”) AND consciousness yields a mere 2.530 results (~10% of the number of papers with a detection task). Our contribution to the literature is thus to show that detection tasks have such a strong biasing effect that even their mere temporal proximity can contaminate predominantly perceptual measures of conscious experience with non-perpeptual effects (rather than claiming a general superiority of reproduction as a perceptual measure over all other methods).

More specifically, a major issue concerns the role of parameters in reproduction tasks that include no-stimulus trials. Over the course of a reproduction experiment, there is an inherent implicit detection component that varies with the proportion of no-stimulus trials, the presented stimulus strengths, and, importantly, the frequency of non-zero responses. These factors can confound what is considered “conscious perception.” This issue should be discussed explicitly and in more depth, including reference to the limits of the reproduction approach itself.

Upon re-reading this statement by the reviewer, we believe a crucial aspect of our study may have been misunderstood, possibly due to the way we described the tasks and how they were (or were not) combined in the different experiments. We are genuinely grateful to the reviewer for bringing this to our attention, as we agree that readers might otherwise arrive at the same interpretation. To clarify, we show that the inclusion or exclusion of stimulus-absent trials is not the cause of the “inherent implicit detection component” during reproduction, and we apologize if we had not explained this sufficiently clearly in the original manuscript. We show in our experiments that the crucial factor that causes the biasing effect of the implicit detection component is the presence of a detection task intermixed with a reproduction task, not the inclusion of no-stimulus (target absent) trials. This becomes apparent when comparing the results from Experiment 1 and 2 to the results from Experiment 3. In Experiment 1 and 2, participants performed both the reproduction task (50% of the trials) and the detection task (50% of the trials), randomly intermixed within the same block. Which of these tasks participants had to perform was indicated by a cue which appeared at the end of each trial. In these experiments, reproductions were clearly biased by the presence of the detection task, as they scaled with the criterion effect in the detection task, as the reviewer correctly points out (also see Figures 2, 3 and 4). However, in Experiment 3 we selectively removed the detection task, so there was no alternation between

tasks anymore, participants *only* had to perform the reproduction task. Crucially, our manipulations of the proportion of target absent (no-stimulus) trials were kept identical in Experiment 3 compared to Experiment 1 and 2. The only thing that changed in Experiment 3 was the removal of the detection task. Removing the detection task abolished the biasing effect of the number of target absent trials on reproduction (base-rate effect), removing biases in reproduction compared to the control condition. Note that the control condition had an equal proportion of target present and target absent trials, whereas the "bias-to-present condition" contained only 10% target absent trials. This is directly illustrated by comparing Figure 5B to Figure 4A and 2C.

This crucial finding shows that the mere presence of a detection task (rather than the proportion of target absent trials) causes biases in reproduction. Given the reviewer's comments, we now understand that we had made this insufficiently clear. For example, we now realize that usage of the phrase "mere presence of a detection context" is ambiguous and confusing, because "detection context" could potentially refer both to varying the proportion of target absent trials or to the presence of an explicit detection task. To mitigate this, we have gone through the entire manuscript to identify references to "the presence of a detection context" and replaced these with "the presence of a detection task". Futher, we now extensively discuss the limitations of the reproduction task in the introduction (lines 102-120) and throughout (see also responses to R1). We hope this clarifies, but we are of course open to making further modifications if needed.

Statements suggesting that traditional methods cannot disentangle perceptual and non-perceptual effects, but reproduction tasks with no-stimulus trials do, may overstate the certainty of the conclusions. Concerns about detection paradigms are well known. The statement "This highlights the need for caution in using and interpreting results relying on detection judgments, despite their central role in consciousness research" seems to overemphasize caution for detection paradigms, contrasting them with reproduction paradigms. Similar caution may be warranted when interpreting results of reprduction tasks, including zero responses in these tasks, as they can also reflect implicit decision criteria rather than the absence of perceptual experience per se.

The abstract and a few sections in the manuscript remain somewhat difficult to follow for a general readership. For example, the connection between the "mere presence of a detection context" and attentional cues, priors, or payoff structures is not immediately clear. Phrases such as "truly experience" are vague. Additionally, simplifying the language throughout and avoiding overcomplicated expressions would enhance readability.

In accordance with the previous point, we have made a number of modifications to clarify our main claim. We never intended to claim that the reproduction task (either with or without no-stimulus trials) is better able to disentangle perceptual from non-perceptual effects than traditional methods. Inclusion or exclusion of no-stimulus (target-absent trials) is not the critical factor. Rather, we claim that the reproduction task without a nearby detection task is better able to disentangle perceptual from non-perceptual effects (regardless of the proportion of target-absent trials in the reproduction task). In our experiments, we show that the presence of a detection task

is the primary culprit, not the proportion of target-absent trials. This thus supports our statement involving “the need for caution in using and interpreting results relying on detection judgments, despite their central role in consciousness research”. Further, we are not tied to the notion that reproduction tasks in themselves are superior. A different kind of appearance-based task can fulfil the same goal, as long as observers in an experiment are not forced to enter “detection mode” by combining such tasks with a detection task (by either intermixing with a detection task, or by presenting both tasks on every trial). We have therefore removed the statement about the superiority of the reproduction task from the abstract, as it is not important for our main claim.

Finally, we fully agree (and show in our manuscript) that reproduction too can be biased by non-perceptual effects. To clarify this in the manuscript, we have added dedicated paragraphs in the Introduction and throughout the text to explicitly acknowledge that reproduction too can be subject to non-perceptual influences (see also our response to R1), and further show that the task set of having to detect stimuli in particular causes such non-perceptual criterion related influences, which disappear when the detection task is removed and reproduction is used in isolation (as in Experiment 3). Finally, to address remaining issues with respect to comprehension for a general readership, we have gone over the entire manuscript again, moderating and clarifying some of our broader claims and improving wording throughout, to avoid overgeneralization and confusion, as well as to improve readability. We hope these changes address the reviewer’s concerns and prevent any misleading interpretation of our claims.

Reviewer #3 (Remarks to the Author):

The authors have sufficiently addressed my comments in this revision. I find the manuscript, suitable for publication.

We thank the reviewer for their feedback on the previous iteration of the manuscript and their positive response to our revision.

Dear Editor,

Thank you for the opportunity to revise our manuscript. We have carefully revised the manuscript according to the editorial request and comments provided by the reviewers. We believe we have incorporated all the suggestions into the manuscript.

Below we provide a document with detailed point-by-point responses to reviewers' comments referring to the locations in the manuscript where changes have been made. For convenience we also provide a document that highlights all the changes that were made relative to the original submission (tracked_changes.pdf).

Thank you for your time and consideration, we hope the revision now meets the requirements for publication in Nature Communications.

Sincerely,

Nicolás Sánchez-Fuenzalida
Chris Jungerius
Stephen M. Fleming
Simon van Gaal
Johannes J. Fahrenfort

Reviewer #1 (Remarks to the Author):

The authors have done a remarkable job revising the manuscript. I only have one remaining comment, aimed at avoiding unnecessary confusion. When discussing serial dependence, the authors now make it a relatively central point in the Introduction, but some statements remain unclear. For example, "The repulsive effect in orientation reproduction suggests that previous decisions can have non-perceptual effects on a subjective measure like reproduction," appears to conflict with earlier arguments that interpret the repulsive effect as perceptual. Given that the nature of both attractive and repulsive serial dependence effects is still actively debated, I would avoid emphasizing this distinction in terms of processing stages too strongly.
My initial comment, which I still consider important, was that reproduction responses are not a direct measure of perception per se, as they are susceptible to multiple post-perceptual influences. These include post-perceptual components of serial dependence effects, but also memory-related biases, category boundary effects, regression to the mean, and other decision-level factors. I think the relevant section could be reduced to this more general point, while still generally referring to serial dependence as an example where post-perceptual factors plays a role.

We would like to thank the reviewer for his or her kind words, and their comments on previous versions that have markedly improved the manuscript. Thank you also for this useful comment. While reading it, we realized we had inadvertently used "repulsive" in this sentence, where we had intended to "attractive". We much appreciate the suggestion to reduce this paragraph to point to a broader set of post-perceptual effects, while referring to serial dependence as a specific case where post-perceptual factors seem to play a role. We have now incorporated this suggestion into the updated manuscript and resolved any unclarities (lines 90-114).

Reviewer #2 (Remarks to the Author):

The changes clearly improved the manuscript. Experiment 3 shows very few non-zero reproductions in the target-absent trials, suggesting that the bias is indeed absent or weak under these specific conditions. However, an implicit detection component is likely still present. It is unclear whether different experimental parameters would allow this component to influence reproduction responses, even in the absence of an explicit detection task, for example with different proportions of zero/non-zero responses, with other parameters varied, or other stimuli/ stimulus dimensions. While the authors now address these limits and refer to future work that would need to replicate these findings, it would be helpful to provide a clear rationale for why the same effect should be expected under other conditions, and not be limited to the specific design, stimuli, and procedure used here.

We would like to thank the reviewer for their constructive comments in improving the manuscript. As for this last comment, Experiment 3 shows that the proportion of zero/non-zero responses in reproduction is identical despite a large difference in the proportion of targets (50% vs 90%). The rationale for why the same effect should be expected under other conditions is that we show that the cause of the base rate effect in reproduction in Experiment 1 and 2 was criterion setting, which we removed by removing the detection requirements in

Experiment 3. We explain this in the discussion and elsewhere. Of course we cannot be 100% sure that there might be circumstances under which reproduction-without-detection might also become biased by base rate in combination with other parameter variations, or by other effects altogether. Indeed, we acknowledge this in the manuscript, and point to some potential non-perceptual biases in the introduction (see also response to Reviewer 1 above). To be sure, our main claim in this manuscript is not that reproduction is always free from post- or non-perceptual effects whenever removing detection requirements (we had already removed any suggestions to that effect), but rather that detection requirements – even if they are not part of the current trial - are a strong catalyst of non-perceptual criterion effects, even on subjective/appearance based measures like reproduction.
